# Spatio-Temporal Gene Expression Profiling during *In Vivo* Early Ovarian Folliculogenesis: Integrated Transcriptomic Study and Molecular Signature of Early Follicular Growth

**DOI:** 10.1371/journal.pone.0141482

**Published:** 2015-11-05

**Authors:** Agnes Bonnet, Bertrand Servin, Philippe Mulsant, Beatrice Mandon-Pepin

**Affiliations:** 1 INRA, UMR 1388 GenPhySE (Génétique, Physiologie et Systèmes d’Elevage), F-31326 Castanet-Tolosan, France; 2 Université de Toulouse, INP, ENSAT, GenPhySE (Génétique, Physiologie et Systèmes d’Elevage), F-31326 Castanet-Tolosan, France; 3 Université de Toulouse, INP, ENVT, GenPhySE (Génétique, Physiologie et Systèmes d’Elevage), F-31076 Toulouse, France; 4 INRA, UMR1198 Biologie du Développement et de la Reproduction, F-78350 Jouy-en-Josas, France; University of Nevada School of Medicine, UNITED STATES

## Abstract

**Background:**

The successful achievement of early ovarian folliculogenesis is important for fertility and reproductive life span. This complex biological process requires the appropriate expression of numerous genes at each developmental stage, in each follicular compartment. Relatively little is known at present about the molecular mechanisms that drive this process, and most gene expression studies have been performed in rodents and without considering the different follicular compartments.

**Results:**

We used RNA-seq technology to explore the sheep transcriptome during early ovarian follicular development in the two main compartments: oocytes and granulosa cells. We documented the differential expression of 3,015 genes during this phase and described the gene expression dynamic specific to these compartments. We showed that important steps occurred during primary/secondary transition in sheep. We also described the *in vivo* molecular course of a number of pathways. In oocytes, these pathways documented the chronology of the acquisition of meiotic competence, migration and cellular organization, while in granulosa cells they concerned adhesion, the formation of cytoplasmic projections and steroid synthesis. This study proposes the involvement in this process of several members of the integrin and BMP families. The expression of genes such as *Kruppel-like factor 9* (*KLF9*) and *BMP binding endothelial regulator* (*BMPER*) was highlighted for the first time during early follicular development, and their proteins were also predicted to be involved in gene regulation. Finally, we selected a data set of 24 biomarkers that enabled the discrimination of early follicular stages and thus offer a molecular signature of early follicular growth. This set of biomarkers includes known genes such as *SPO11 meiotic protein covalently bound to DSB* (*SPO11*), *bone morphogenetic protein 15* (*BMP15*) and *WEE1 homolog 2* (*S*. *pombe*)(*WEE2*) which play critical roles in follicular development but other biomarkers are also likely to play significant roles in this process.

**Conclusions:**

To our knowledge, this is the first *in vivo* spatio-temporal exploration of transcriptomes derived from early follicles in sheep.

## Introduction

In large mammalian species at birth, the ovaries contain a large and fixed reserve of non-growing primordial follicles (oocytes surrounded by flattened pre-granulosa cells). Most of these follicles remain in the resting state until either their degeneration or their activation and growth towards the primary, secondary and tertiary stages (with an antral cavity). The gradual exit of primordial follicles starts shortly after formation of the primordial follicle pool and continues throughout the reproductive years [1]. This early follicular development is therefore important as it regulates the size of the remaining stock of primordial follicles and their fate, which in turn affects fertility and the reproductive life span.

Early follicular development is accompanied by an increase in oocyte diameter, a progressive acquisition of competence [2] and proliferation of granulosa cells (GCs). Early follicular development requires the appropriate expression of numerous genes at different developmental stages and orchestrated communication between the two main compartments (oocytes and granulosa cells) [3, 4]. These compartments regulate follicle growth in an autocrine and paracrine manner via secreted factors and direct gap junctional communication.

Therefore, any integrated study of folliculogenesis must include changes in gene expression in all cell types, but such studies are faced with two major problems: restricted access to isolated stages and the limited supply of RNA. Indeed, the presence of all follicular stages in the ovary and the small size of preantral follicles, render the isolation of each early follicular stage even more problematic. As a result, relatively little is known about the key molecular mechanisms that underlie the complex biological processes of *in vivo* early folliculogenesis.

Until now, some transcriptomic studies focused on primordial-primary transition from neonatal rodent ovaries on day 0–2 and day 4 that were respectively enriched in primordial and primary follicles [5, 6], or from pure populations of oocytes from human primordial, intermediate and primary follicles [7]. Only two mouse studies explored the breadth of early follicular development from oocytes and whole follicles, respectively [8, 9]. They showed that the principal change in expression in mouse species occurs at oocyte primordial-primary transition [8]. They revealed an over-representation of differentially expressed genes involved in protein synthesis and the cell cycle, in particular M-phase, throughout the early growth phase [8]. The differential expression of oocyte-secreted ligands involved in NOTCH, SHH, EGF, TGFβ, PDGF signaling pathways [8, 9] also suggests that multiple signaling pathways operate during follicular development. Nevertheless, these results obtained in poly-ovulating species cannot be fully exploited in mono-ovulating species (sheep, human), so recent studies addressed this issue in the latter. A first study focused on secondary and small antrum follicles in goat species and highlighted three main metabolic pathways: lipid, cell death and hematological pathways [10]. However, our previous studies had been the first to describe differential gene expression between two isolated compartments, granulosa cells and oocytes, in sheep ovarian follicles [11, 12]. We identified enriched functional categories that reflect two distinct cell fates. We revealed the involvement of granulosa cell pathways such as SHH, WNT and RHO GTPase. Other signaling pathways such as VEGF, NOTCH and IGF1 suggested the existence of complex cell-cell interactions. The expression and the specificity of the two cell types are now characterized in sheep species [11].

Nevertheless, until now no studies had been able to describe the gene expression changes that occur during early follicular growth and in each compartment of the follicle. Consequently, the aim of the present study was to: 1- focus and decipher gene expression and functions that might be associated with early folliculogenesis from a developmental point of view (from primordial, primary, secondary and small antrum follicles) in sheep species, and 2- produce a set of biomarkers that are able to predict early follicular growth and could be used as *in vivo* signatures of early folliculogenesis.

For this purpose, we used the previous dataset of 15,349 gene expressions obtained by RNA-seq technology [12] in order to analyze the spatio-temporal gene expression. We were able to show that major changes in gene expression occur during the primary-secondary transition. We compiled a detailed description of the molecular mechanisms underlying early folliculogenesis in sheep which are probably transposable to other large mammals including humans.

## Materials and Methods

### RNA-seq experiment

This study was carried out in strict accordance with the institutional guidelines for research studies. The protocol was approved by the prefecture de la Haute Garonne (animal experimentation authorization no 31–297).

We briefly described the dataset previously published [11]:

- The lambs were euthanized at birth (<1 day). The ovaries were removed and embedded in O.C.T. embedding matrix, frozen in liquid nitrogen and stored at -80°C until use. Frozen sections were fixed and stained before laser capture microdissection [11].
- The follicular compartments (*i*.*e*. granulosa cells (GCs)) and oocytes (O) were selected for each follicular stage as a function of follicular diameter (primordial (PD<35μM), primary (PM: 35–50μM), secondary (SC: 60–120μM) follicles and small antral follicles (SA: 250–500μM), captured by laser capture microdissection and extracted using the PicoPure RNA Isolation Kit. Three/four independent replicates were obtained per sample.
- Thirteen tissue samples (fetal ovary, pituitary gland, hypothalamus, muscle, skin, heart, lung, intestine, stomach, kidney, spleen, liver, and theca) were collected in triplicate (from three separate animals) at the local slaughterhouse for RNA extraction. The 12 total RNA tissue samples (not including fetal ovary) were pooled in three separate groups.
- A total of 31 RNA LCM-derived (3 oocyte samples from primordial follicles (PDO), 4 oocyte samples from primary follicles (PMO), 4 oocyte samples from secondary follicles (SCO), 4 oocyte samples from small antral follicles (SAO), 4 granulosa samples from primordial follicles (PDG), 4 granulosa samples from primary follicles (PMG), 4 granulosa samples from secondary follicles (SCG) and 4 granulosa samples from small antral follicles (SAG)) and three multi-tissue RNA samples (3 independent pools of 12 total RNA tissue samples) were subjected to two rounds of T7 linear amplification.
- Sequencing was performed on an Illumina HiSeq2000 using the Illumina TruSeq SBS kit v2 (209 cycles including the index) to obtain paired-end reads (2x100 base pairs (bp)).
- The reads were mapped with bwa aln to the sheep genome sequence [13] (CSIRO Oarv2.0 released March 2011: http://www.livestockgenomics.csiro.au/sheep/). Overlapping reads were merged to produce genomic fragments.

The gene annotation of genomic fragments was performed by alignment with the Bos taurus UMD3.1 genome using Blat and filtered to conserve only the best genomic fragment for each gene.

### Statistical analysis

The significance of differential gene expression was determined using the DESeq package [14] of R software for multi-factorial design (R 2.14.0; DESeq release 1.6.1). Our experimental design included two factors: “stage” (with four levels: PD, PM, SC and SA) and “compartment” (two levels: O, GCs) [12]. To establish the differential expression profile throughout early follicular development we identified the genes whose intra-compartment variance was differentially expressed during at least one of the stages (nbinom GLM Test). For this, we specified two models: the stage model (count ~ stage) and the reduced model (count ~ 1) and applied them to each compartment dataset. For each compartment, the expression value of each gene was then compared between each follicular stage using pairwise comparison (nbinom Test). Last, differential gene expression during early follicular development was selected with a FDR <5% for the global intra-compartment effect (nbinom GLM Test) and pval <1% for the pairwise comparison (nbinom Test) with a fold change >2.

### Quantitative real-time PCR analysis of gene expression

Gene primer designs were based on the RNA-seq sequences as previously described [12] (S1 Table).

The LCM-derived aRNA samples were reverse transcribed as previously described [11]. The reactions were completed to 50μl and diluted at 1/20 prior to PCR. The assay for each gene consisted of four replicates per condition (except for PDO = 3) and negative controls. Gene expression was analyzed using 96.96 Dynamic Array™ IFCs and the BioMark™ HD System from Fluidigm, as previously described [11].

The PCR amplification efficiency of genes was determined from a foetal ovary cDNA pool (11 ng cDNA) that was serial diluted (1, 1:3; 1:3; 1:2; 1:2).

After determining the threshold cycle (Ct) for each LCM-derived aRNA sample, the PFAFFL method was applied to calculate the relative expression of each gene [15]. This relative expression was normalized using the corresponding geometric average of three reference genes with geNorm v3.4 [16]: *β-actin*, transmembrane p24 trafficking protein 4 *(TMED4)* and ribosomal protein L19 (*RPL19)* genes that were respectively slightly, moderately and highly expressed and not regulated during follicular development or in the compartment.

The significance of the relative expression data on genes involved in canonical pathways was tested using the one-way ANOVA model of the R statistical software system (the Comprehensive R Archive National, http://www.cran.r-project.org) after logarithm transformation of the data. For each gene, an ANOVA model was fitted using two factors: “stage” (4 levels) and “compartment” (2 levels) and their interactions. A backward variable selection procedure was applied, as previously described [11].

### Biological trends

Ingenuity^®^ Pathway Analysis software (IPA; http://www.ingenuity.com) was used to examine the functional enrichment in differentially expressed genes. The significance of the association between the differential gene lists and functional categories was determined by a P value calculated using Fisher’s exact test corrected for multiple testing (FDR <0.05 (Benjamini-Hochberg test)).

Pathway based analysis was performed using Webgestalt (http://bioinfo.vanderbilt.edu/webgestalt/ Kegg, Wikipathway and Pathwaycommons databases) and GeneCodis (http://genecodis.cnb.csic.es/ Kegg pathway and Panther databases) software programs. Significantly enriched pathways were identified using a hypergeometric test followed by a multiple testing correction of p-values using the Benjamini-Hochberg test (FDR <0.05). Then, to ensure that pathways obtained from computational biology were coherent and gave a good interpretation of experimental results [14, 17], only Webgestalt significant pathways identified using the two softwares (Webgestalt and GeneCodis) or from two databases, were conserved (Kegg/Wikipathway/ Pathwaycommons databases).

IPA downstream Effects Analysis was then used to identify the expected effects of our observed gene expression changes on the functions (expected to increase or decrease). This analysis examined genes in our dataset that were known to affect functions, compared the directions of change of the genes with those expected based on findings in the literature, and then issued a prediction for each function based on the direction of change (in the differential gene expression list).

In the same way, IPA Upstream Regulator analysis was used to understand the reasons for the gene expression changes observed. It identified the cascade of upstream transcriptional regulators that might explain the gene expression changes observed in the dataset and predicted the activity of their encoded protein (cf. Methods section). Taking account of the gene expression profile (expressed and/or differentially expressed) for each predicted upstream regulator provided more evidence for the biological mechanism. The expression and/or differential expression of the transcriptional regulator in the dataset produced more evidence for a particular biological mechanism. These analyses were described by Bonnet et *al*. [12].

### Biomarker selection

Biomarkers of the compartments and stages of early folliculogenesis were determined using the DESeq package [14] of R software for multi-factorial design (R 2.14.0; DESeq release 1.6.1). The genes mostly expressed in a specific cell type and stage (PDO, PMO, SCO, SAO, PDG, PMG, SCG, SAG) were selected using pairwise comparisons (nbinom Test) with other samples, including multi-tissue samples (FDR<5% and a fold change >3 for granulosa samples and a fold change >10 for oocytes samples (for example, PDO / PMO + SCO + SAO + PDG + PMG+ SCG+SAG + MT)). Theca cells were included in multi-tissue samples to ensure the selection of specific expression in oocytes or granulosa cells and to prevent contamination.

The accuracy of biomarker selection was checked using qRT-PCR and the significance of differential gene expression was evaluated using Student's test after a fourth square transformation.

PLS regression was used to classify and discriminate the samples (PLS-DA). PLS-DA was implemented using the mixOmics package under R software.

### Linear mixed models for expression data

The linear mixed models were described in S1 Text. Briefly:

#### Logistic regression model for the presence/absence of expression

We first of all estimated a model based on the binary response and indicating the expression or lack of expression, with respect to the gene, developmental stage, cell type and replicate. This was modeled with a hierarchial logistic regression, including correlation parameters between random effects of different stages. All random effects were found to be highly significant (*p*<10^−7^).

#### Linear regression on the quantitative level of expression

Secondly, we estimated a model on the level of expression for gene, developmental stage, cell type and replicate using a fourth-root transformation of the raw expression. In cases where no expression was found in any replicate, we included a single observation with null expression in the data.

#### Equations for the prediction of developmental stage given a vector of expression data

Given the above models estimated on the RT-PCR data, we derived equations that could predict the stage of the follicle, given the cell type and a vector of expression data on the genes. Specifically, we were dealing with a situation where we were given a vector of new observations (binary response and expression level), for a given cell type, and wanted to predict the stage reached by the corresponding follicle.

#### Predictive ability of the models

To test the predictive ability of the models, we implemented the predictive equations using a customized program and generated new vectors for observations through re-sampling: for a given cell type and for each stage, we generated a new data vector with one observation for each gene by sampling one value taken at random from the observed data. Thus each vector was a new set of observations drawn from the original data. We performed this re-sampling method 100 times for each stage. We then applied the predictive equations to each of the 100 vectors and recorded the posterior probabilities of each stage.

## Results and Discussion

### Global differential gene expression during early follicular development

This study was carried out using the gene profiles of 15,349 genes generated by RNA-seq experiment (http://www.ebi.ac.uk/arrayexpress/ under accession number E-MTAB-1587) [12]. This experiment used a laser capture microdissection method to split and produce granulosa cells or oocyte transcriptomes for each stage of early follicular development: primordial (PD), primary (PM), secondary (SC) and small antral (SA) follicle stages. The experiment included three/four biological replicates per sample. Previous study applied a generalized linear model (DESeq GLM) to this dataset to explore the differential expression and the specificity between each compartment (oocyte, granulosa cells). We also observed 19.6% of genes that were significantly differentially expressed (DEG) during early development (3,015 genes, FDR<5%) [12].

In this study, we specifically investigated the 3,015 DEG (S2 Table) in order to clarify the dynamics of transcription during early development and we observed more marked changes in gene expression in oocytes than in granulosa cells (2173/1192). A majority of the differentially expressed genes were down-regulated during early folliculogenesis in granulosa cells (66%), while the numbers of up- and down-regulated genes were similar in oocytes (Table 1).

**Table 1:** Summary of differential gene expressions.

	Number of differential genes		
	Primary stage	Secondary stage	Small antrum stage
oocyte	173	1005	2074
granulosa cells	171	732	1074

**Fig 1:**
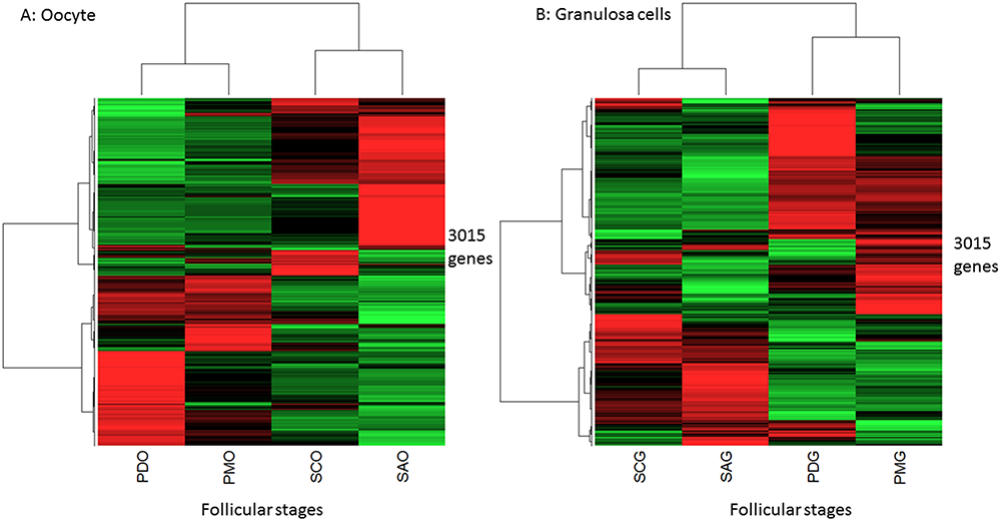
Heatmap display of unsupervised hierarchical clustering of all differential genes during early folliculogenesis. This Figure shows two unsupervised hierarchical clusterings from oocyte (A) and granulosa cells (B) differential gene expressions. The first level of the dendrogram classified the primordial/primary follicles and the secondary/small antrum follicles as two distinct clusters. The other levels revealed a lower dissimilarity between primordial/primary than small antrum/secondary stages. The genes are shown in lines and the relative expression means by follicle stages are shown in columns. Red, black and green represent the levels of expression: up, mean, and down, respectively. PDO: oocyte from primordial follicles, PMO: oocyte from primary follicles, SCO: oocyte from secondary follicles, SAO: oocyte from small antrum follicles, PDG: granulosa cells from primordial follicles, PMG: granulosa cells from primary follicles, SCG: granulosa cells from secondary follicles, SAG: granulosa cells from small antrum follicles.

An unsupervised hierarchical clustering was performed to explore differential expression and confirmed the relevance of these data (Fig 1). The branching distance of the first level of each dendrogram revealed a marked dissimilarity between primordial/primary follicles and secondary/small antrum follicles. The distance branching of the second and third levels of the dendrogram highlighted a clear separation between the other follicular stages. These clusterings depict the dynamics of expression during early follicular development and suggested important differential expression between primary and secondary stages.

In term of differential gene expression between two consecutive stages (S1A and S1B Fig), a weak differential expression could be seen during primordial/primary transition. The differences in gene expression between stages mainly started during primary-secondary transition, when 74% and 67.4% of differential genes displayed decreased expression in oocytes and granulosa cells, respectively. The number of differentially expressed genes was then maintained during oocyte secondary/small antrum transition (476 genes in the primary/secondary stages and 494 genes in the secondary/small antrum stages) whereas few differential gene expressions were found in granulosa cells during secondary/small antrum transition: 55 genes (S1A and S1B Fig).

These results therefore described a difference in expression dynamics between the two compartments during early development, with 1- important and constant changes in gene expression in oocytes that were in line with the extremely rapid growth of oocytes prior to the formation of antrum [18], and 2- fewer changes affecting granulosa gene expression, which was in line with the fact that most granulosa cell proliferation and differentiation occurs later during the antral phase when the oocytes have almost stopped growing [18]. Lastly, contrary to mouse species in which primordial/primary transition has been identified as the major transition [8], in sheep species we were able to highlight major changes of expression during primary/secondary transition (Fig 1 and S1 Fig).

### Validation of differential expression

Consistent with previous reports, our list of differentially expressed genes (3,015 genes) included genes known for their differential expression during early follicular development (for example *BMP15*, *growth differentiation factor 9* (*GDF9)*, spermatogenesis and *oogenesis specific basic helix-loop-helix 2* (*SOHLH2)*, *NLR family pyrin domain containing 5* (*NLRP5)*). In addition, the expression profiles of 19 genes of interest involved in enriched canonical pathways (BMP, IGF1, Gap junction, WNT, FGF, PI3K, RAR activation, apoptosis.) were monitored using qRT-PCR, and statistical analysis confirmed the DE observed in the RNA-seq dataset for 14 of them (S3 Table).

### Global outlook relative to gene functions and pathways

Six global lists of differentially expressed genes (taking account of pairwise comparisons) were created to explore significant functions and pathways involved in early folliculogenesis (oocyte lists (PMO/PDO, SCO/PDO, SAO/PDO) and granulosa cell lists (PMG/PDG, SCG/PDG, SAG/ PDG)).

#### Biological functions

**Fig 2:**
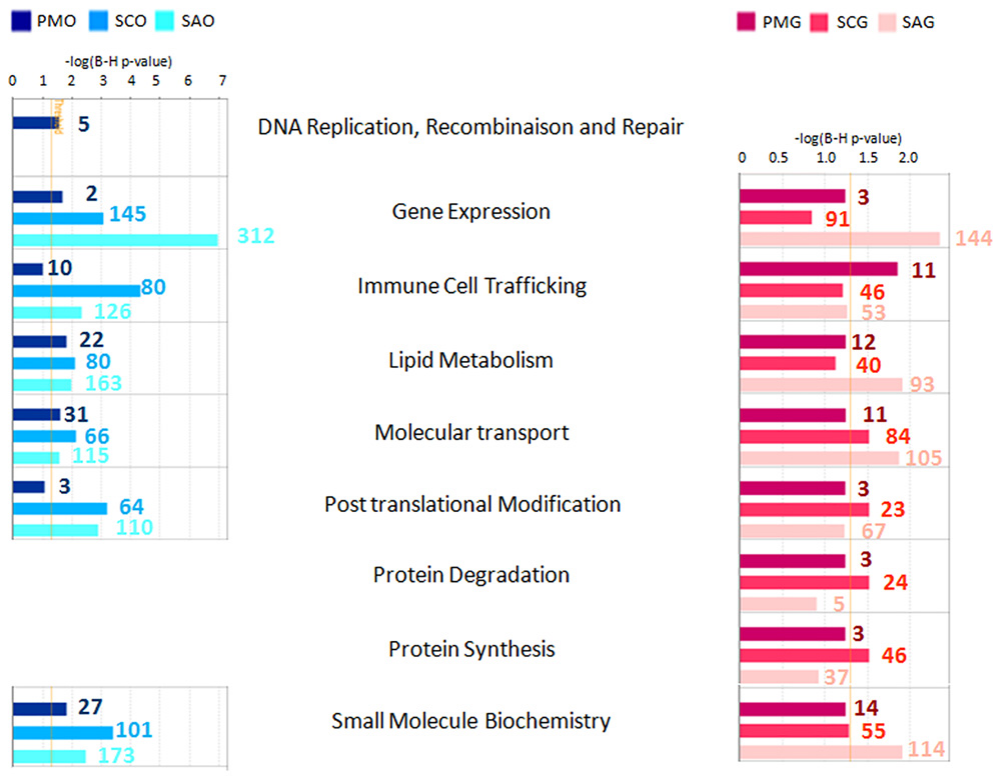
Cellular function enrichment. Functional enrichment analysis for sets of differentially expressed genes during early follicular development was performed *in silico* for each compartment using Ingenuity Pathway Analysis (IPA) software. Statistical significance was determined by a P value calculated using Fisher’s exact test corrected for multiple testing correction (Benjamini-Hochberg test, FDR <0.05). This analysis identified nine main cellular function categories as being significantly enriched in differentially expressed genes. Bar colors correspond to enriched functions at primary stage (compared to primordial stage), secondary stage (compared to primary and primordial stages), and small antrum stage (compared to secondary, primary and primordial stages). The X axis corresponds to the level of significance of the function: -log(B-H p value). Granulosa cell functions are colored in red and oocyte functions are colored in blue. Numbers correspond to the number of focus genes contributing to the functions. Follicular stage abbreviations are described in Fig 1.

The biological functions associated with early follicular development were investigated using Ingenuity Pathway Analysis (IPA). Ten functional categories and nine main cellular function categories were identified as being significantly enriched in differentially expressed genes (FDR<0.05) and are presented respectively in S2 Fig and Fig 2.

At the primary stage, the DNA replication, recombination and repair category was specifically enriched in differentially oocyte expressed genes. These genes are essential to protecting genetic information during meiosis. Their corresponding proteins repair copy errors and block the resumption of meiosis. In the granulosa cell compartment, we observed the expression of several genes which were also involved in this functional category (DNA replication, recombination and repair category) but they did not change during follicular development. This granulosa cell gene expression stability was in agreement with a slow follicular grow process (130 days in sheep species) and a weak rise in the granulosa cell mitotic index up to early antral follicles in either sheep (from 60 μm (secondary follicles: 831 h doubling time) to 280 μm of follicular diameter (early small antrum follicles: 244 h doubling time)) [19] or human species [20]. Intense granulosa cell proliferation mainly occurred later during antral stage (from 280 μm follicular diameter up to 850 μm in sheep species) and was supported by a rapid rise in the mitotic index [19].

At the secondary stage, IPA analysis highlighted major functional change and two biological functions relative to protein metabolism (degradation, synthesis) as being mainly over-represented in the secondary stages of granulosa cells but not enriched in differentially expressed genes during oocyte development.

#### Pathways

**Fig 3:**
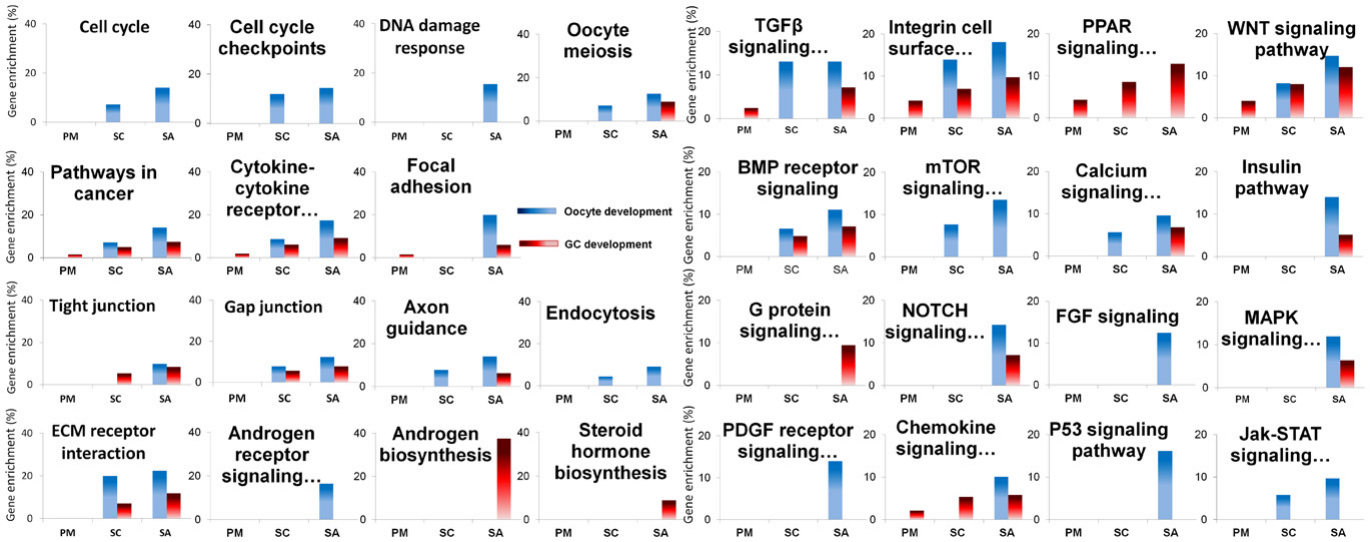
Significantly enriched pathways highlighted in early folliculogenesis. Pathway enrichment analysis for sets of differentially expressed genes during early follicular development was performed using Webgestalt software. Statistical significance was determined by multiple testing correction of p-values using Benjamini-Hochberg test (FDR <0.05). Thirty one enriched pathways are presented. Each graph corresponds to the percentage of gene enrichment in the pathway (%) according to different stages of follicles (PM (primary), SC (secondary) and SA (small antrum)). Granulosa canonical pathways are colored in red and oocyte canonical pathways are colored in blue.

Pathway based analyses were investigated using Webgestalt software and revealed significant pathway enrichment in the lists of differentially expressed genes (FDR<0.05; S4 Table). These pathways encompassed cell processing, signaling pathways and those involved in cell communication that were slightly enriched in primary follicles and mainly enriched from the secondary stage when compared to primordial stage (Fig 3). However, some pathways were only significantly enriched at the small antrum stage.

At the primary stage, we noted a slight enrichment in regulated genes involved in cell dialog such as cytokine-cytokine receptors and integrin cell surface interactions, chemokine signaling, focal adhesion and TGFB and WNT signaling. The PPAR signaling pathway, which is known to be involved in lipid metabolism, was specifically enriched in granulosa cells.

At the secondary stage, we underlined the enrichment of genes involved in oocyte cell cycle and meiosis (*cyclin-dependent kinase 1* (*CDK1)*, *cyclin B1* (CCNB1), *Moloney sarcoma oncogene* (*MOS)*, *TTK protein kinase (TTK)*, *etc*…) (Fig 3). We also observed pathways that were related to physical and molecular communication such as endocytosis, tight and gap junctions, ECM receptor interaction and neuronal signal transmission (axonal guidance). Fig 3 shows the enrichment of additional signaling pathways such as the BMP receptor signaling network, mTOR, calcium and Jak-STAT signaling pathways.

At the small antrum stage, we identified an enrichment of genes involved in the oocyte DNA damage response and granulosa cell steroid hormone signaling processes (Fig 3). Finally, members of the PDGF receptor family (such as *platelet derived growth factor D (PDGFD)*, *platelet-derived growth factor receptors (PDGFRA* and *PDGFRB)*, *etc*…) and members of FGF signaling pathways (*fibroblast growth factor 1 (FGF1)*, *neural cell adhesion molecule 1* (*NCAM1*), *syndecan 2* (*SDC2)*, *etc*…) were regulated in oocytes of small antrum follicles. Members of the G protein signaling pathway were regulated in granulosa cells while the insulin, NOTCH, and MAPK signaling pathways were enriched in differential genes in both the oocyte and granulosa cells compartments (S4 Table).

#### Downstream effects

In addition to the above studies, we used IPA to predict the downstream effect of differentially expressed genes on the functions (expected to be activated or inhibited). Apart from the categories linked to cell growth, this analysis showed that numerous categories were affected (S5 Table) which participated in cell communication and organization, cell growth and lipid metabolism.

Together the data on the principal processes in play, these analytical findings now offer an overview of early follicular development.

### Biological interpretation

At the end of the preantral stage, a large quantity of mRNA is stocked in oocytes which then become competent to resume meiosis. Moreover, granulosa cells may synthesize estrogens and respond to gonadotropin hormones.

The different IPA results (functions (Fig 2; S2 Fig), pathways (S4 Table), downstream effects (S5 Table)) were compiled to describe the molecular processes involved in achieving this status.

#### Chronology of meiotic maturation

**Fig 4:**
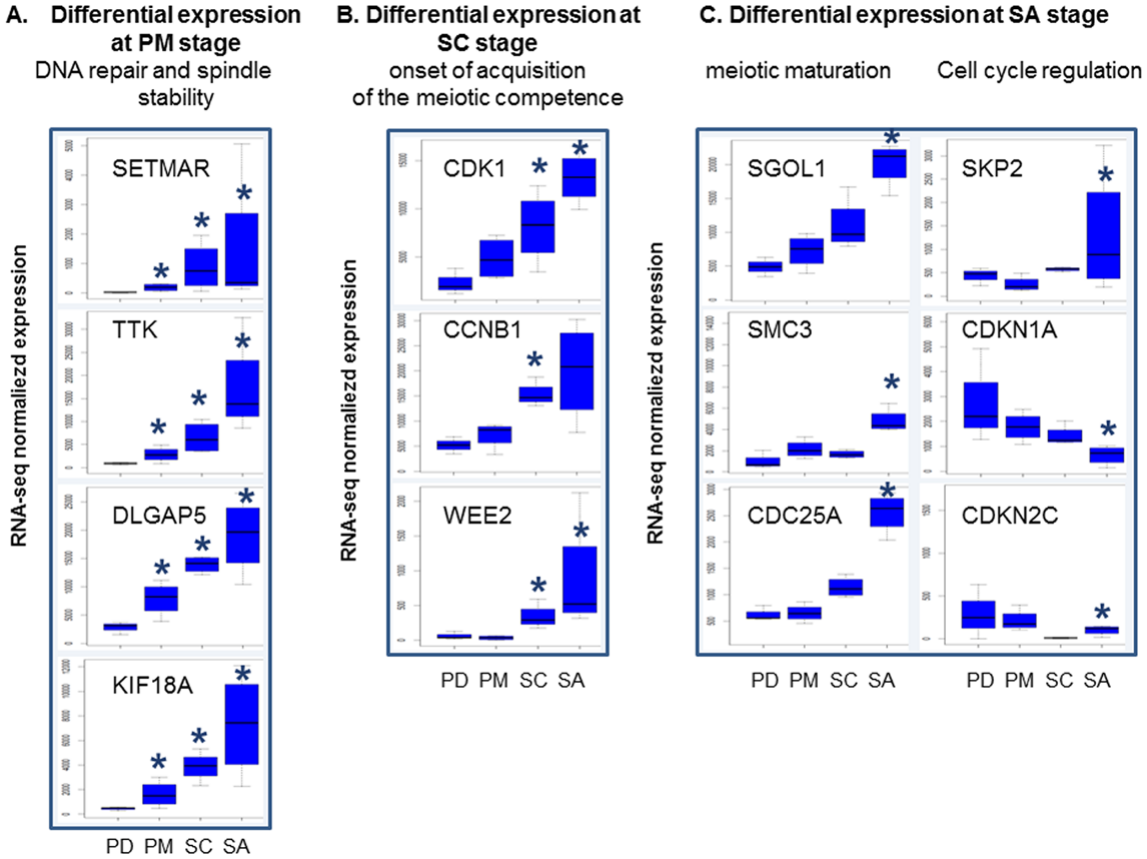
Meiotic maturation gene expression as a function of oocyte developmental stage. (A) Differential gene expression at primary stage, (B) differential gene expression at secondary stage, (C) differential gene expression at small antrum stage. The Y axis corresponds to normalized counts from RNA-seq data. *: FDR <0.05, pairwise comparison pval <0.01.

Meiotic maturation is essential for the correct formation of metaphase II oocytes, and is mainly acquired during early folliculogenesis [21]. This complex process depends on the interplay in time and space between the cytoskeleton (spindle stability) and the cell cycle machinery, and on the coordinated exchange of signals with somatic cells. During this study, we identified a chronology in the expression of genes involved in the regulatory network governing the maintenance of meiotic arrest and the acquisition of meiotic competence (Fig 4).

During the primordial to primary transition, we observed an increase in the expression of genes including *SET domain and mariner transposase fusion gene (SETMAR)*, *TT*K, *discs*, *large (Drosophila) homolog-associated protein 5* (*DLGAP5)* and *kinesin family member 18A (KIF18A)*, *etc*., which are involved in the DNA replication, recombination and repair (Fig 4A). Thus, for the first time, we are able to identify an ovarian expression of the *SETMAR* gene (Fig 4A) that was shown to promote double strand break (DSB) [22] and non-homologous end joining (NHEJ) [23] repairs in human lineage cells. Its over expression in oocyte of primary follicles, by comparison with primordial follicles, is in line with the role of the protein to maintain genomic stability. TTK, DLGAP5 and KIF18A regulate the cell cycle by controlling chromosome alignment, spindle assembly and attachment. TTK (or MPS1) is a spindle assembly checkpoint protein that is necessary for chromosome segregation during oocyte meiosis I [24]. DLGAP5 stabilizes kinetochore fibers and promotes chromosome alignment on the spindle. Kinesins play essential roles in assembling spindles, separating centrosomes and attaching chromosomes to spindles [25] and could cause female sterility [26]. In Hela cells or regenerating mouse liver, the *DLGAP5* mRNA levels were found to be tightly regulated during cell cycle progression. In particular, the level of expression was elevated during the G2/M phase [27]. Consequently, the over-expression of *DLGAP5* mRNA in ovine oocytes from primary follicles accorded with their cell cycle arrest at the G2/M phase. Its protein also regulates KIF18A localization [28] whose the gene was highlighted for the first time as differentially expressed during primordial/primary transition (3.62 fold change).

At the secondary stage, we identified an increased expression of genes such as *CDK1* and *CCNB1*, the constituents of oocyte maturation promoting factor (MPF), as well as the meiosis inhibitor *WEE2* [29] (S4 Table, cell cycle; Fig 4B). MFP governs the G2/M transition of the cell cycle and must remain inactive to ensure the arrest of meiosis. The ability to resume meiosis is acquired when an oocyte obtains a sufficient amount of MPF. Our findings were in agreement with the initiation of acquisition of meiotic competence already described in mice at the secondary stage [8].

At the small antral stage, the acquisition of meiotic competence progressed (cell cycle, cell cycle checkpoints and oocyte meiosis pathways; S4 Table). The progression in each phase of the cell cycle is governed by cyclin-dependent kinases (Cdks) within Cyclin-Cdk complexes. These Cdks are negatively regulated by small polypeptides from the Cip/Kip and INK4 families such as *cyclin-dependent kinase inhibitor 1A* (CDKN1A /p21/cip1) and *cyclin-dependent kinase inhibitor 2C* (CDKN2C/p18INK4c), respectively [30]. Our data point out this cell cycle regulation with for example the over-expression of *S-phase kinase-associated protein 2*, *E3 ubiquitin protein ligase* (*SKP2)* that is involved in the degradation of CDKN1A [31], and then the down expression of *CDKN1A* (S4 Table, cell cycle; Fig 4C). *CDKN2C* gene also displayed an interesting temporal expression profile with decreased expression during primary/secondary transition and increased expression during secondary/small antrum transition. This gene profile illustrated the fine regulation of oocyte meiosis arrest during oocyte development. This profile accorded with the fact that the *CDKN2C* gene may function in a cell type-specific [30] and differentiation-specific manner [32]. Last we observed the over-expression of genes necessary to stabilize mouse oocyte meiotic arrest such as *cell division cycle 25A (CDC25A)* [33] or to maintain the fidelity of chromosome segregation (*shugoshin-like 1 (S*. *pombe)* (*SGOL1)*, *structural maintenance of chromosomes 3* (*SMC3)*; S4 Table, oocyte meiosis).

#### Cell-cell communication

Follicular growth is dependent not only on close interactions between oocytes and granulosa cells but also between granulosa cells themselves. These interactions include molecular dialog and physical communications [12].

During oocyte development, the migration and cellular organization categories were affected by changes in gene expression.

At the primary stage, IPA highlighted genes involved in cell migration that might be associated with follicle activation in the growing phase (S5 Table; migration of endothelial cells: increased expression of *adrenomedullin (ADM)*, *calcitonin-related polypeptide alpha (CALCA)*, *thyroglobulin (TG)*, *gastrin-releasing peptide (GRP)* genes and decreased expression of inhibitor of *DNA binding 3*, *dominant negative helix-loop-helix protein* (*ID3)*, *protein kinase D1* (*PRKD1)*, *secreted frizzled-related protein 4* (*SFRP4)* genes). For example, *ADM* [34] and *CALCA* [35] genes are known to regulate cell proliferation and migration in vascular endothelial and vascular smooth muscle cells.

At secondary and small antrum stages, the number of differentially expressed genes involved in cell migration increased (S5 Table; migration of cells: 182 differential expression genes (DEG)). For example, genes related to cell migration signaling pathways such as integrins (*integrin alpha M (ITGAM)*, *integrin beta 8* (*ITGB8)*, *caveolin 1* (*CAV1)*, etc.), WNT (*transforming growth factor*, *beta receptor II* (*TGFBR2)*, *SFRP4*, *wingless-type MMTV integration site family member 5A* (*WNT5A)*, etc.) or Rho (Fig 3; S4 Table) were mainly down-regulated.

During follicular development and at the secondary stage, granulosa cells exhibited an interesting subset of differentially expressed genes that was concerned with the adhesion (S5 Table; adhesion of blood cells: 25 DEG) and the formation of cytoplasmic projections (S5 Table; formation of neurites: 12 DEG). Lastly, a number of regulated genes encode for proteins displaying cell contact (*neural precursor cell expressed*, *developmentally down-regulated 9* (*NEDD9)*, *secreted phosphoprotein 1 (SPP1)*, *integrin alpha 5–6* (*ITGA5-6)*; S5 Table; adhesion of blood cells) and chemokine properties as kit ligand *(KITL) (* S5 Table; cytokine-cytokine receptor interaction).

Last, these results offer further evidence to reinforce the role of WNT and integrin signaling pathways in cell-cell communication during early follicular development (Fig 3).

**Fig 5:**
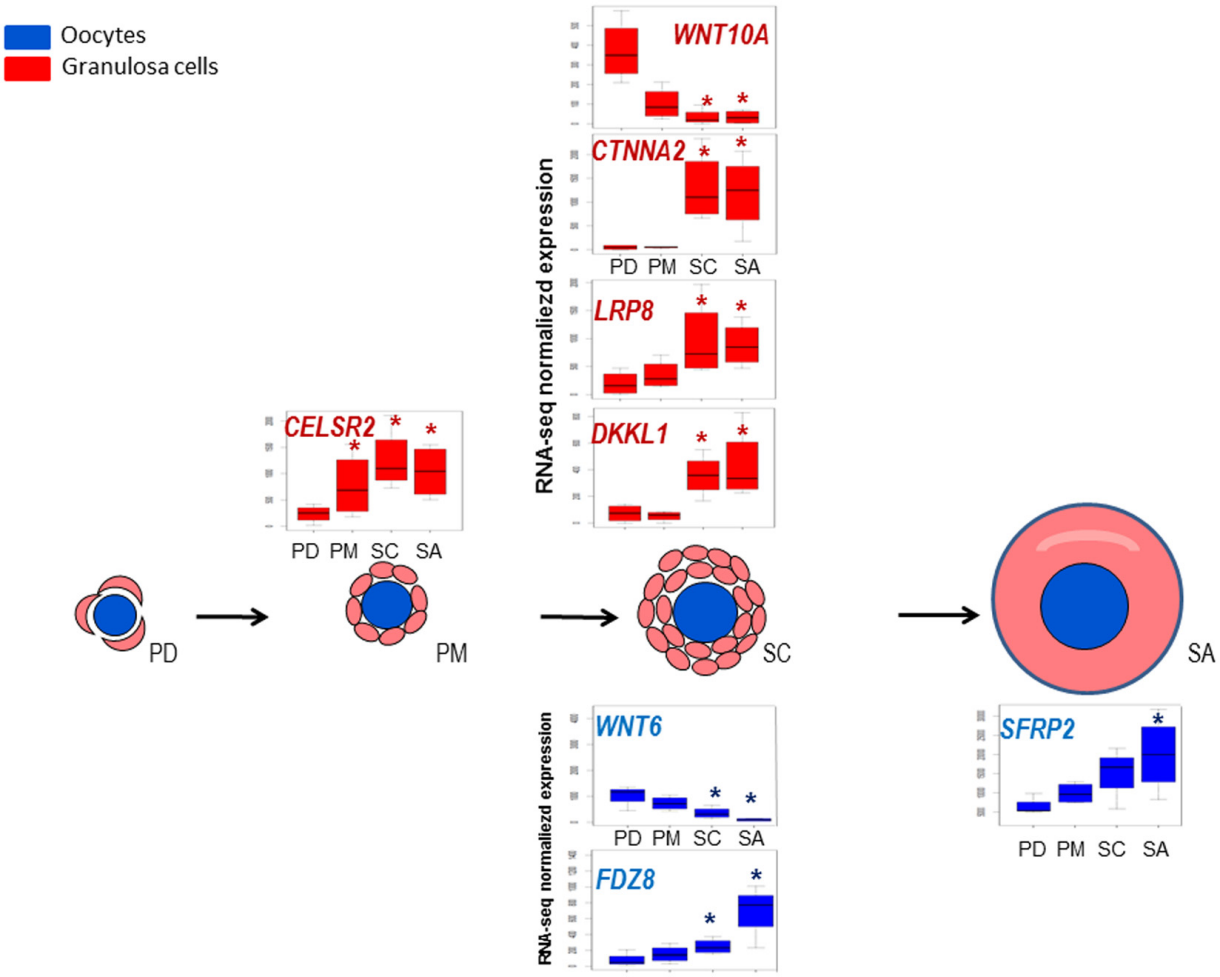
Gene expression profiles of members of WNT signaling pathway during early folliculogenesis. Significant differences in relative gene expression during early folliculogenesis of members of Wnt signaling pathway (S2 Table). The Y axis corresponds to normalized counts from RNA-seq data. Granulosa cells data are colored in red and oocyte data are colored in blue. *: FDR <0.05, pairwise comparison pval <0.01.

The expression of antagonists that regulate the WNT pathway, such as *dickkopf-like 1* (*DKKL1)*, *dickkopf WNT signaling pathway inhibitor 1* (*DKK1)* and *secreted frizzled-related protein2* (*SFRP2)*, increased during preantral folliculogenesis. We also identified the granulosa cell over expression of *cadherin*, *EGF LAG seven-pass G-type receptor 2* (*CELSR2)* from primary follicles and two additional regulating genes from secondary follicles as *catenin alpha 2 (CTNNA2)*, *low density lipoprotein receptor-related protein 8* (*LRP8)* (S5 Table). CELSR2 is an atypical cadherin, that is able to transduce signals from the non canonical WNT/PCP signaling pathway [37] and plays an important role the control of tissue polarity in planar cells [38]. CTNNA2 is a catenin isoform that links cadherin adhesion receptors. LRP8 is a positive regulator of Wnt/β-catenin signaling that increases Wnt-induced transcriptional responses [36]. Gene expression profiles are presented Fig 5.

**Fig 6:**
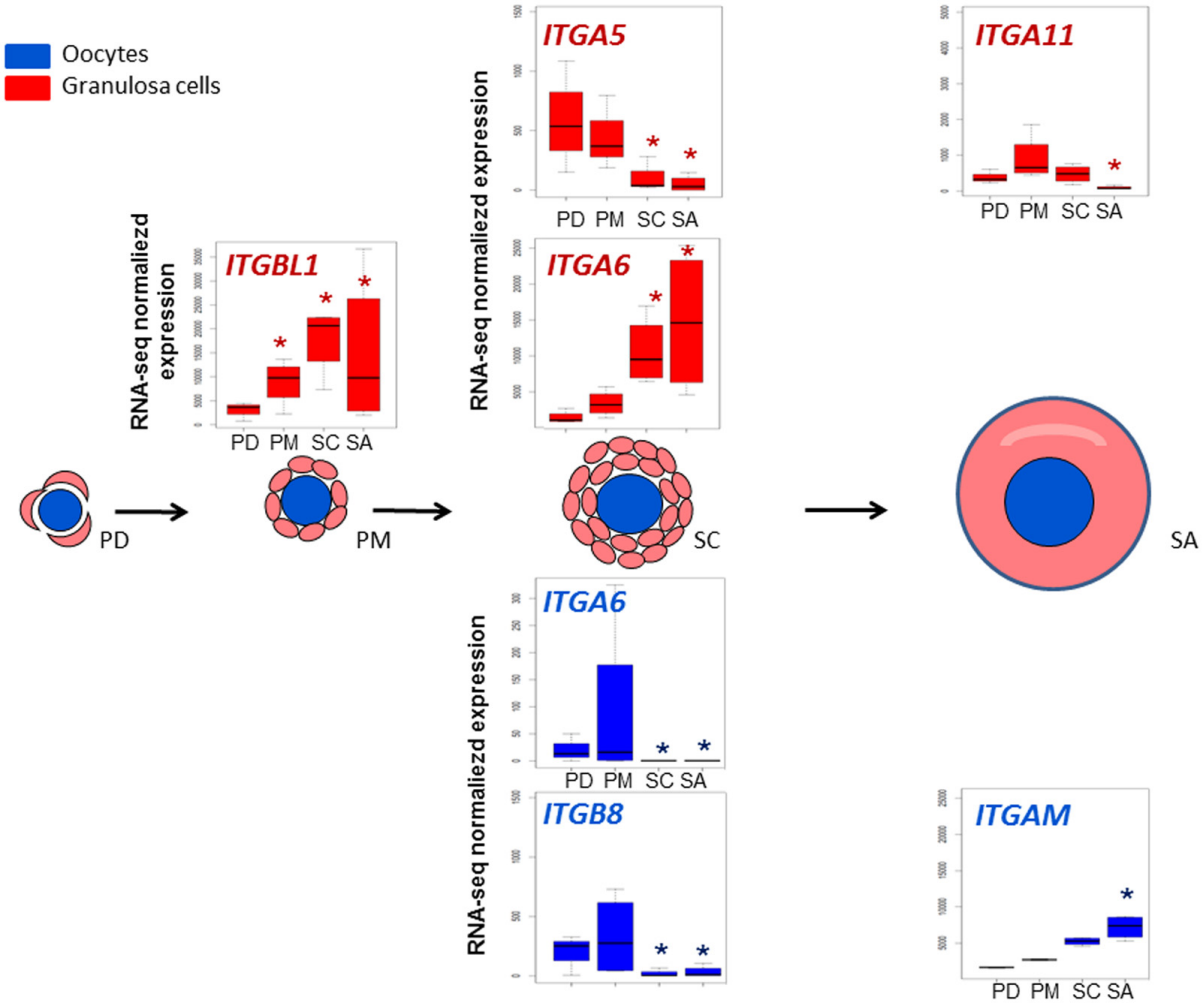
Dynamic representation of integrin family gene expression. Significant differences in relative gene expression during early folliculogenesis of members of integrin family (S2 Table). Granulosa cells data are colored in red and oocyte data are colored in blue. The Y axis corresponds to normalized counts from RNA-seq data. *: FDR <0.05, pairwise comparison pval <0.01.

In addition to the regulated integrin family genes identified during oocyte development (*ITGAM*, *ITGB8)*, we noted the regulation of other genes of this family during follicular progression such as *ITGA5-6* and *integrin beta-like 1* (*ITGBL1)* (Fig 6). *ITGA5* expression has never been described in the ovary. *Itga6* is expressed in the granulosa cells of early follicles in the mouse [39]. In marmoset species, atretic tertiary follicular status is associated with a lack of *ITGA6* and *ITGB1* expression [40]. Our study showed that the expression of *ITGA6* increased at the secondary stage whereas that of *ITGA5* decreased. In the same way, *ITGBL1* increased. In accordance with marmoset data, *ITGA6* expression profile suggests that *ITGA6* may promote cell contact in order to preserve follicle health during early folliculogenesis in sheep.

#### Granulosa cell steroid synthesis

**Fig 7:**
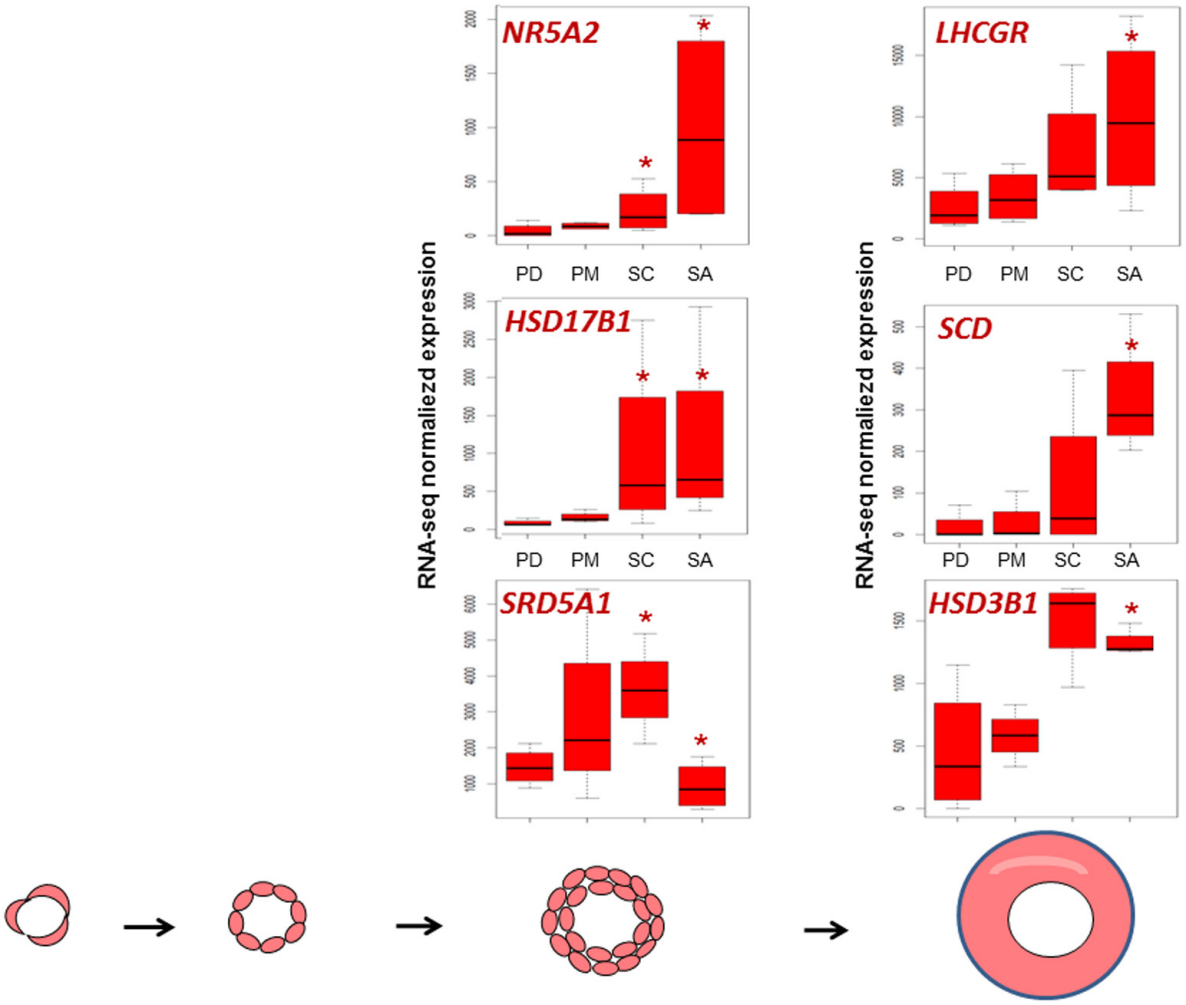
Dynamic representation of gene expression involved in steroid biosynthesis process. Significant differences in relative gene expression during early folliculogenesis of genes involved in steroid synthesis (S2 Table). *: FDR <0.05, pairwise comparison pval <0.01.

At the end of early follicular development, granulosa cells may be capable of producing steroids. Pig preantral follicles produced hormones such as estradiol and progesterone [41] that originate from cholesterol and are required for the development and maturation of follicles [42]. We demonstrated a gradual increase during follicular development in the expression of genes already known to participate in lipid and steroid biosynthesis (Fig 3). At the small antral stage granulosa cells over-expressed the *stearoyl-CoA desaturase* (*SCD)* gene that is involved in lipid metabolism which had previously been observed as being weakly expressed in rat primordial follicles and mainly expressed in granulosa cells of antral follicles [43]. Other differential genes concerned steroid synthesis such as *steroid-5-alpha-reductase*, *alpha polypeptide 1* (*SRD5A1)*, *hydroxy-delta-5-steroid dehydrogenase*, *3 beta- and steroid delta-isomerase 1 (HSD3β)* and *hydroxysteroid (17-beta) dehydrogenase (HSD17β)* and *steroid regulation such as the nuclear receptor subfamily 5*, *group A*, *member 2 (NR5A2)* [44] and *luteinizing hormone/choriogonadotropin receptor* (*LHCGR)*. In line with these changes, IPA predicted an increase in the steroid biosynthesis process (S5 Table). Gene expression profiles are summarized in Fig 7.

### Putative regulators involved during early folliculogenesis

IPA Upstream Regulator analysis was used in order to understand the cause of the gene expression changes observed. The relevance and usefulness of this analysis (S6 Table) were confirmed by the identification of important molecules and pathways known to affect or be involved in follicular growth. Indeed, our analysis confirmed the important roles of NOBOX oogenesis homeobox (NOBOX), KITL and PDGF at key stages in early development.

**Fig 8:**
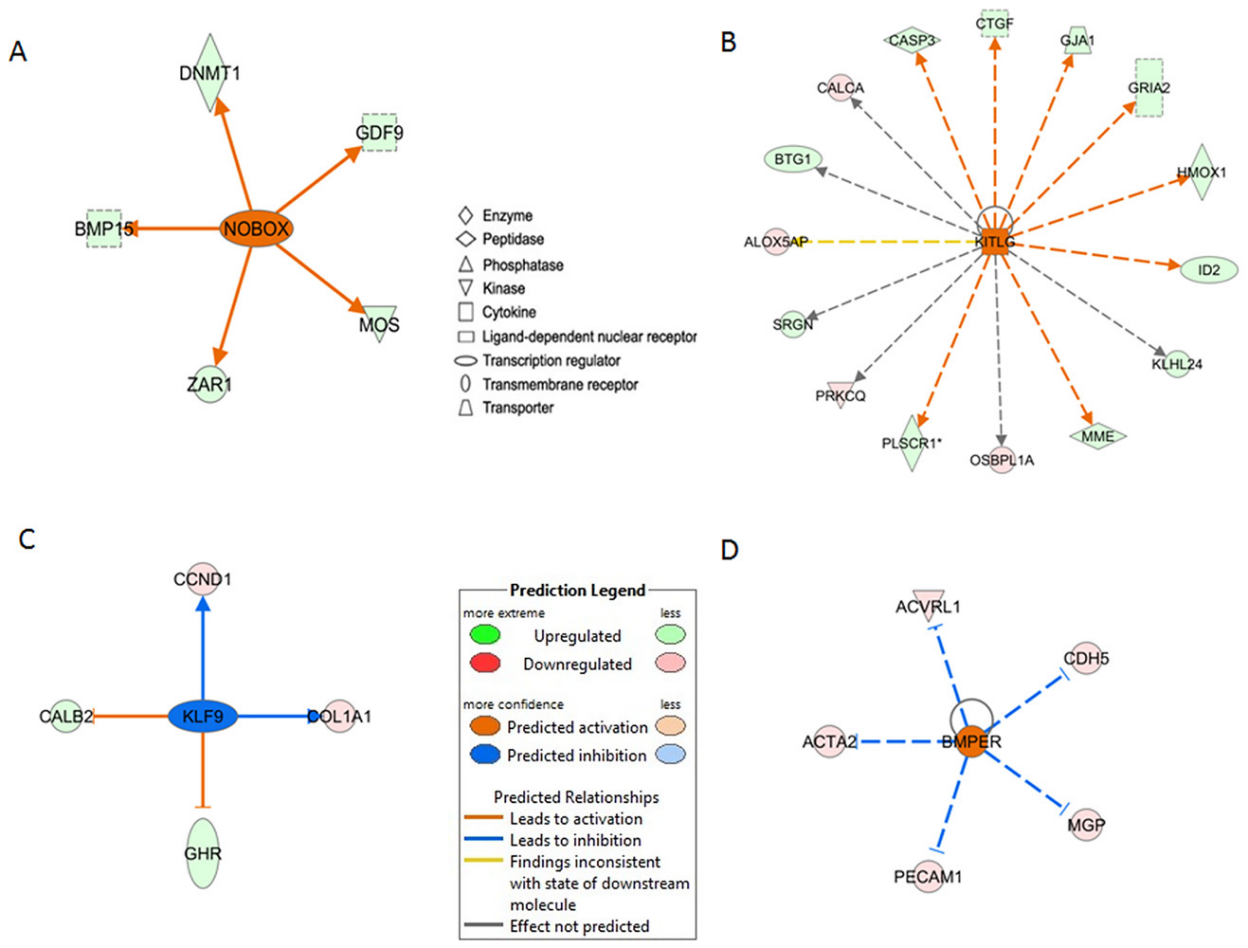
Upstream regulators involved during early folliculogenesis. This Figure focuses on four upstream regulators of interest predicted to be activated or inhibited at key transitions during follicular development. Genes in our dataset are highlighted based on up- (green) or down- (red) expression and increasing intensity in line with the degree of fold change. The predicted action of the central gene is indicated as activated (orange) or inhibited (blue) with the degree of confidence increasing in line with color intensity. Arrowheads at the ends of the interactions indicate activation, whereas bars indicate inhibitory effects. Unbroken arrows and dashed arrows represent direct and indirect interactions between genes and upstream regulators, respectively. Predicted activation of (A) NOBOX in oocytes at small antrum stage, (B) KITL in granulosa cells at small antrum stage, (C) KLF9 in oocytes at small antrum stage, (D) BMPER in oocytes at secondary stage.

Genes that were differentially expressed at small antral stage enabled prediction of an increased activity of the NOBOX protein in ovine oocytes and of KITL protein in ovine granulosa cells (Fig 8A and 8B), and a decreased activity of PDGFC in oocytes. In mice, Nobox activates expression of the *BMP15*, *DNA (cytosine-5)-methyltransferase 1* (*DNMT1)*, *GDF9*, *MOS*, *zygote arrest 1* (*ZAR1)* genes in oocytes [45]. In our experiment, sheep oocytes exhibited an over-expression of the *BMP15*, *DNMT1*, *GDF9*, *MOS*, *ZAR1* genes at small antral stage when compared to the primordial stage and a stable expression of NOBOX during early folliculogenesis. This analysis therefore suggests an increase in NOBOX protein activity (Fig 8A). KITL protein acts at different levels of follicular development [46]. During early folliculogenesis, this protein promotes GC mitogenesis [47] via indirect mechanisms that may involve GDF-9 [3] and BMP-15 [48]. In agreement with these studies, we identified in ovine granulosa cells four down-expressed genes and 11 over-expressed genes known to be regulated by KITL (Fig 8B). In line with these changes, our RNAseq data also showed a significant over expression of *KITL* during follicular development. Last, we showed that *PDGFC* and *PDGFRA-B* are mainly expressed in granulosa cells (S2 Table) as has already been demonstrated [9], and are less expressed in oocytes. The predicted reduction in the action of PDGFC protein on oocytes at small antrum stage was in line with a decrease in *PDGFC* granulosa cell expression and *PDGFRA* and *PDGFRB* oocyte expression during early folliculogenesis in sheep (S6 and S4 Tables).

Two molecules that have been the subject of little study in the context of follicular development were predicted to significantly affect transcription during early folliculogenesis: KLF9 and BMPER (Fig 8C and 8D; S6 Table). Studies have shown that KLF9 stimulates *cyclin D1* (*Ccnd1)* [49] and *collagen*, *type I*, *alpha 1* (*Col1a1)* [50] expression and inhibits that of *calbindin 2* (*Calb2)* and *growth hormone receptor (Ghr)* [51]. During our experiment, we observed the opposite regulation of these genes. As a result, IPA predicted an inhibited status for the KLF9 protein (Fig 8C). In line with this, *KLF9* gene expression decreased during early follicular development. KLF9 protein is involved in a large number of functions, including reproduction. It modulates the sensitivity of endometrial cells to oestradiol [52]. In pig granulosa cells, KLFs (KLF4-9-13) may recruit inhibitory complexes containing HDAC co-repressors in order to repress the transcription of *low density lipoprotein receptor (LDLR*) and *cytochrome P450*, *family 11* (*CYP11)* [53]. The role of KLF9 in oocytes still needs to be elucidated. Lastly, our findings suggest the involvement of BMPER as an oocyte regulator during primary transition to secondary follicles (Fig 8D). BMPER has been reported to antagonize the activities of BMP2-4-3-6 [54] and displays significant affinity with another BMP antagonist, chordin (CHRD) [55]. This gene is poorly documented in the ovary. In mice, the *bmper* gene is expressed from the primary follicles but is not detected in oocytes [56]. This gene is described here for the first time as being mainly expressed in sheep oocyte and up-regulated from the secondary stage (S2 Table). The downstream oocyte targets of BMPER are *actin alpha 2* (*ACTA2)*, *activin A receptor type II-like 1* (*ACVRL1)*, *matrix Gla protein* (*MGP)* and *platelet/endothelial cell adhesion molecule 1* (*PECAM1)*, the expression of which decreased at the secondary stage in our study (Fig 8D). Further experiments are now necessary to understand the role of BMPER in folliculogenesis. In addition, we identified the differential expression of other signaling BMP antagonists in granulosa cells or oocytes such as *follistatin (FST)*, WAP, *follistatin/kazal*, *immunoglobulin*, *kunitz and netrin domain containing 2 (WFIKKN2)* and *gremlin 2 (GREM2)* (S3 Fig), thus highlighting the complex regulation of the BMP signaling pathway during early folliculogenesis.

### Biomarkers of early folliculogenesis

#### Biomarker selection

**Fig 9:**
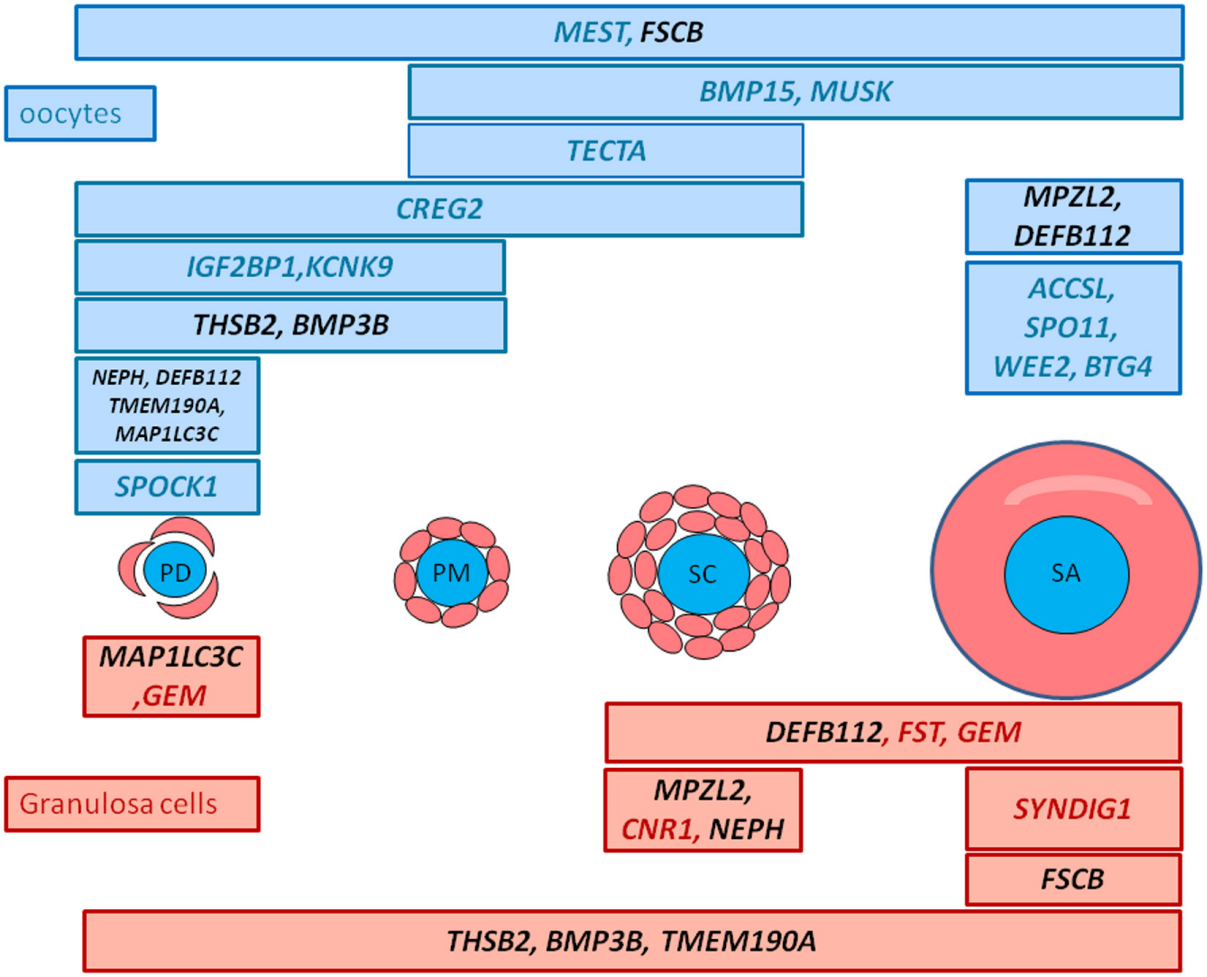
Distribution of the expression of the 24 biomarkers during early follicular development. These biomarkers correspond to the most strongly expressed genes in a specific cell type and stage (PDO, PMO, SCO, SAO, PDG, PMG, SCG, SAG). They were selected using pairwise comparisons (nbinom Test) comparing each stage to the others including multi-tissue samples (FDR<5% and a fold change >3 for granulosa samples and a fold change >-10 for oocyte samples). Their differential expression was confirmed by qRT-PCR. Gene names marked in red are genes expressed in granulosa cells and gene names marked in blue are genes expressed in oocytes. Gene names marked in black are genes expressed both in oocytes and granulosa cells.

A number of protocols have been developed for domestic species such as cattle, buffalo and sheep regarding the performance of *in vitro* functional analysis, manipulation and embryo production, or the evaluation of cryopreservation prior to cancer treatments. The identification of biomarkers is therefore an important step because they can be used to monitor follicular development and survival. For this purpose, we performed a pair-wise comparison between each stage dataset and the other datasets including multi-tissue samples (FDR<5%, FC>3–10) and selected the 29 most differentially expressed genes (see Materials and Methods). The expression of this gene selection was then checked by qRT-PCR. Statistical analysis confirmed the differential expression observed in the RNA-seq data for 24 out of the 29 genes tested (S7 Table). Lastly, the two sets of 24 expression profiles arising from RNAseq and RT-PCR were subjected to a PLS-DA analysis to evaluate its capacity to discriminate follicular stages (S4 Fig). SPLS-DA analysis is a partial least squares regression technique applied to categorical variables (here the stages of follicles). PLS-DA has often been used for classification and discrimination or variable selection. As expected, these two analyses (RNA-seq/qRT-PCR) gave similar discrimination of the follicle stages, making this biomarker set easily usable by qRT-PCR technology. The two analyses showed some structure of variables without clearly separating the stages. Consequently we turned to using parametric models for the difference in expression levels across stages, and specifically the ability of our biomarkers to predict the follicular stage. A summary of the 24 significantly differential profiles is presented in Fig 9.

#### Predictive ability of biomarkers

Based on these RT-PCR expression data, we estimated linear mixed models of expression that could be explained by developmental stage and cell type in the 24 differentially expressed genes. We then used these estimated models to derive predictive equations of stage conditional on the expression data for each cell type (oocyte and granulosa cells). Details of these procedures are given in the Methods section.

The expressions of these genes were modeled jointly using linear mixed models. These data displayed both a binary nature (differences in the presence/absence of expression across stages and cell types) and a quantitative nature (different levels of expression). This behavior was clarified using two different models: 1- a model for the presence/absence of expression using a hierarchical logistic regression model, and 2- hierarchical linear regression to determine the quantitative level of expression.

Both models included parameters to fit gene and cell type effects within stages relative to either the probability (model 1) or quantitative level (model 2) of expression. With model 1, we found that across the genes, the probability of expression at the PD, PM and SC but not SA stages were highly correlated (>0.99). Within cell types, the correlations between the PD and PM stages on the one hand, and the SC and SA stages on the other, were also very high (>0.9). Using model 2, we found that the levels of expression across the genes between the PD and PM stages were highly correlated (>0.99) but that the expression levels between the PD or PM and SA stage were highly negatively correlated (<-0.99).

**Fig 10:**
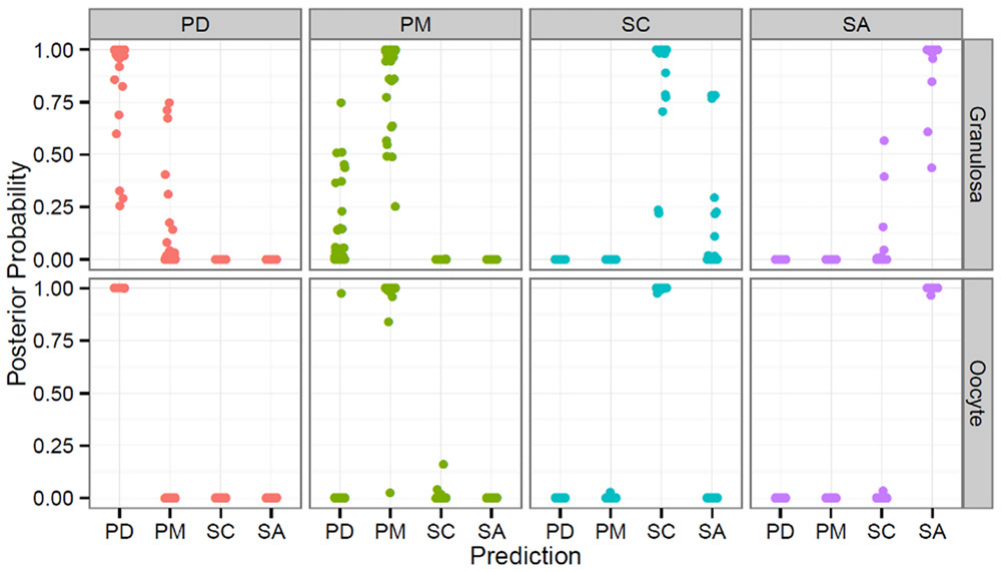
Predictive power of the combination of logistic regression and the linear regression model. Predictive power of biomarkers using linear mixed model equations incorporating both the presence/absence of expression and its quantitative level. The scatter-plot shows the posterior probability that an expression vector arises from each of the possible stages, when the simulated vector is made up of observations from the PD, PM, SC or SA stages (from left to right), for granulosa cells (top) and oocytes (bottom).

Using the estimated models, we then derived predictive equations for the follicular stage from a vector of observed expression levels on all genes. We then simulated 100 new observation vectors from the original data, for each stage (see Methods for details; S1 Text). Fig 10 presents the posterior probability of an expression vector arising from each of the possible stages, when the simulated vector was made up of observations from the PD, PM, SC or SA stages (from left to right), for granulosa (top) and oocyte (bottom) cells. In Fig 10, both the presence and absence of expression, and quantitative expression levels, are taken into account. The predictive ability of the model is very good, with the posterior probability of the correct stage being almost always equal to one for all stages. We noted however that this predictive ability was higher for oocytes than the granulosa cells. We also found that the binary response alone did not ensure good predictive ability for most stages (see S5 Fig). For example, when sampling a vector from the PD stage in oocytes, the posterior probabilities were spread almost evenly between the PD and PM stages.

Our results therefore suggest that our predictive equations could be used to obtain reliable data on the follicular stage of a cell from the expression data for this set of genes. However, we noted that this predictive ability might be over-estimated as the random vectors generated were obtained by random sampling of the data used to estimate the parameters for the predictive equations. Determining this predictive ability on independent data was not possible here, but this issue will be addressed in the context of a future study.

#### Biological relevance of biomarkers

The list of biomarkers shown in Fig 9 includes known genes such as *SPO11*, *BMP15*, *FST* and *WEE2* which play critical roles in meiosis, primary-secondary transition or early folliculogenesis. For example, *BMP15* and *FST* expression profiles indicated cell type and stage-specific gene expressions as previously described [11]. This confirms the level of purity of the LCM-derived aRNA samples, and the quality of our RNAseq analysis. Other biomarkers are also likely to play significant role in follicular development.

At the primordial stage, we highlighted the oocyte expression of *potassium channel*, *two pore domain subfamily K*, *member 9* (*KCNK9)*, s*parc/osteonectin*, *cwcv and kazal-like domains proteoglycan (testican) 1* (*SPOCK1)*, *insulin-like growth factor 2 mRNA binding protein 1* (*IGF2BP1)* and the granulosa cell expression of *microtubule-associated protein 1 light chain 3 gamma* (*MAP1LC3C)*. *KCNK9* encodes a potassium channel family protein that has already been identified in the membrane of cow oocytes [57]. *SPOCK1* encodes for a proteoglycan protein. It was initially identified in human testis and tissue distribution studies have shown that it is most prominently expressed in nervous tissue. It can block the substrate attachment and neurite outgrowth of neuronal cells [58] and may therefore regulate brain development. Until now, no studies have explored *SPOCK1* in the ovary. *IGF2BP1* belongs to a family of RNA-binding proteins implicated in mRNA localization, turnover and translational control. The spatio-temporal expression of IGF2BP1 protein has been described in the female gonads of mouse and humans [59]. Its expression is ubiquitous up to 16.5 days in the mouse embryonic stage and then becomes restricted to germ cells. There is usually no expression in adult tissues, with the exception of gonads. In adult mouse and human ovaries, IGF2BP1 protein has been detected in both resting and growing oocytes and less clearly detected in granulosa cells. *IGF2BP1* mRNA is nevertheless a good marker of primordial oocytes in sheep. The *MAP1LC3C* gene is mainly expressed in granulosa cells of primordial follicles. This gene is involved in the formation of autophagosomal vacuoles, an autophagy activity that is fundamental to many processes across the reproduction spectrum, from development of the primordial follicle and spermatozoa to embryogenesis [60]. For example, autophagy is involved in maintaining the primordial follicles in a dormant state via non apoptotic mechanism [61, 62]. Our results reinforce these findings. Later, at antral stages, it was also shown that autophagy process is also implicated in follicular atresia in different species [63, 64] and may be regulated through cross talk with apoptosis proteins [65].

At the primary stage we identified expression of the *tectorin alpha (TECTA)* gene in sheep oocytes. TECTA protein is one of the major noncollagenous components of the tectorial membrane and plays a role in intracochlear sound transmission. This gene was not documented in other tissues.

At the secondary stage we highlighted the expression of the *cannabinoid receptor 1* (*CNR1)* gene in granulosa cells. This gene encodes one of cannabinoid receptors known to regulate spermatic differentiation. Its invalidation influences sperm chromatin remodelling by reducing the displacement of histones [66]. Its role in granulosa cells still needs to be defined.

Finally at the small antral stage we highlighted the granulosa cell expression of the *synapse differentiation inducing 1* (*SYNDIG1)* gene and the oocyte expression of the *SPO11*, *WEE2* and *B-cell translocation gene 4* (*BTG4)* genes. *SYNDIG1* encodes the SYNG1 protein that belongs to the interferon-induced transmembrane family of proteins. This protein is a regulator of excitatory synapse maturation and induces synapse maturation [67]. Its role during follicular development requires further investigation. Surprisingly, during this study, we identified a preferential expression of *SPO11* in small antrum oocytes. It is assumed that SPO11 is necessary to generate chromatin breaks during leptotene of the first meiotic prophase in yeast and mammals [58, 68]. In early ovarian follicles, this process is completed and germ cell meiosis is arrested at the diplotene stage. The function of *SPO11* after DNA double-strand breaks (DSBs) has been little studied and it would be interesting to investigate its role as from the end of prophase I. Thus Houmard et *al* showed in the human fetal ovary that the number of *SPO11* transcripts was elevated after 12 weeks of gestation (corresponding to the end of meiosis) and remained elevated up to 18 weeks (initiation of follicle formation at 12–14 weeks) [69]. The detection of *SPO11* expression in sheep oocytes of small antrum follicles was in line with this previous human study so we can hypothesize new roles for SPO11 during oocyte development. Wee2 is responsible for phosphorylating the CDK1 inhibitory site and maintaining meiotic arrest in oocytes. In macaque monkeys species, and as seen during our study, *WEE2* mRNA appears to accumulate during folliculogenesis, reaching the highest level in preovulatory follicles [29]. BTG4 belongs to the PC3/BTG/TOB family of cell-cycle inhibitors. It is able to induce G_1_ arrest, and is highly expressed in the testis, oocytes, and preimplantation embryos [70]. *BTG4* is also highly expressed in bovine testis and ovary/oocyte, which suggests a role for BTG4 in meiosis [71, 72].

This biomarker set thus offers an “*in vivo”* reference to predict follicular development by a useable qRT-PCR technology.

## Conclusions

For the first time, a detailed and comprehensive spatio-temporal transcriptome for the developmental stages of early folliculogenesis has been presented in this study. Substantial changes in gene expression occur in both oocytes and granulosa cells as the follicles develop. We have described the specific gene expression dynamic during follicular development for each compartment. We identified more gene expression change in oocytes than in granulosa cells, and revealed important differential expressions between primary and secondary stages in sheep. Gene expression was little affected in granulosa cells at the last studied stage (small antrum stage).

Our analysis highlighted the *in vivo* molecular course of a number of processes. As early as the primary stage, a significant number of genes involved in the cell cycle regulation were found to be differentially expressed in oocytes, illustrating initiation of the acquisition of meiotic competence. Other changes in gene expression affected oocyte migration and cellular organization. At the secondary stage, genes related to cell migration signaling pathways such as integrins, WNT or Rho were mainly down-regulated in oocytes. In neighboring cells (granulosa cells), an interesting subset of genes was found to be involved in the adhesion and formation of cytoplasmic projections. The expression of integrin family members, involved in cell morphology, was notably affected in both compartments. At the small antral stage, the acquisition of meiotic competence progressed with an over-expression of genes required to stabilize mouse oocyte meiotic arrest. We demonstrated an increase in the expression of genes already known to participate in lipid and steroid biosynthesis in granulosa cells. Lastly, some important pathways were found to be associated with early follicular development, such as FGF signaling in oocytes and WNT and BMP signaling in both compartments.

Numerous questions remain regarding the mechanisms governing the regulation of early follicular growth. Our findings proposed the new involvement of a number of molecules such as KLF9 and BMPER in gene regulation during this growth. We also generated a data set of 24 biomarkers that could be useful to predict follicular growth. They also are likely to play important roles in the process of follicular growth. They enabled discrimination between early follicular stages and thus offer a molecular signature for early follicular growth. They are of importance to determining the quality and correct development of *in vitro* or *in vivo* protocols for the study of early folliculogenesis, the production of embryos or estimation of the cryogenic preservation of the ovarian cortex.

This study has therefore highlighted new arguments that enable a better understanding of the molecular mechanisms involved in early ovarian follicular development and new insights into both reproductive biology and regenerative medicine.

## Supporting Information

S1 Fig Analysis of changes in expression during stage transition. This Figure shows the number of differentially expressed genes (under- and over-expressed) in oocyte (A) and granulosa cells (B) at each follicular transition: primordial/primary, primary/secondary, secondary/small antrum.
(TIF)

S2 Fig Functional enrichment during early follicular development. Genes differentially expressed during early development were evaluated *in silico* using Ingenuity Pathway Analysis (IPA) for each compartment (FDR <5%). The bar color corresponds to enriched functions at primary stage (compared to primordial stage), secondary stage (compared to primary and primordial stages), and small antrum stage (compared to secondary, primary and primordial stages). The X axis corresponds to the level of significance of the function: -log(B-H p value). Granulosa cell functions are colored in red and oocyte functions are colored in blue. Numbers correspond to the numbers of focus genes that contributed to the functions.
(TIF)

S3 Fig Gene expression profiles of the genes involved in BMP signaling. Y axis corresponds to normalized counts from RNA-seq data. GC data are colored in red and oocyte data are colored in blue.
(TIF)

S4 Fig (s)PLS-DA analysis of the biomarker set transcriptome. (s) PLS-DA was performed on the biomarker dataset after normalization using the DEseq R package to classify follicular stages according to gene expression. The Figure visualizes the first three components of the analysis from: (A) RNA-seq dataset, (B) qRT-PCR dataset.
(TIF)

S5 Fig Predictive power of the logistic regression model. Predictive power of biomarkers using linear mixed model equations incorporating only the presence/absence of expression. The scatter-plot shows the posterior probability that an expression vector arises from each of the possible stages, when the simulated vector is made up of observations from the PD, PM, SC or SA stages (from left to right), for granulosa cells (top) and oocytes (bottom).
(TIF)

S1 Table Primer sequences for real-time PCR. (XLSX)

S2 Table Genes significantly differentially expressed during early follicular development. (XLSX)

S3 Table QRT-PCR validation. The expression profiles of 19 genes of interest involved in enriched canonical pathways were monitored using qRT-PCR, and statistical analysis confirmed the DE observed in the RNA-seq dataset for 14 of them.
(DOCX)

S4 Table Significantly enriched oocyte and granulosa cell canonical pathways during early follicular development. Significant pathway enrichment in differentially expressed genes was investigated using webgestalt software (FRD<0.05).
(XLSX)

S5 Table Analysis of downstream effects. This analysis predicted the effect of change in gene expression on the functions using IPA software.
(XLSX)

S6 Table Analysis of upstream effects. IPA Upstream Regulator analysis was used to obtain clues regarding the cause of the change in gene expression and to provide more evidence regarding the biological mechanism.
(XLSX)

S7 Table qRT-PCR validation of gene expression in the biomarker set. (XLSX)

S1 Text Description of Linear Mixed models. (DOCX)
