# Supplementary figures and images for: Spatio-Temporal Gene Expression Profiling during In Vivo Early Ovarian Folliculogenesis: Integrated Transcriptomic Study and Molecular Signature of Early Follicular Growth
Source: PLoS One. 2015 Nov 5;10(11):e0141482. doi: 10.1371/journal.pone.0141482 (PMC4634757; doi:10.1371/journal.pone.0141482)

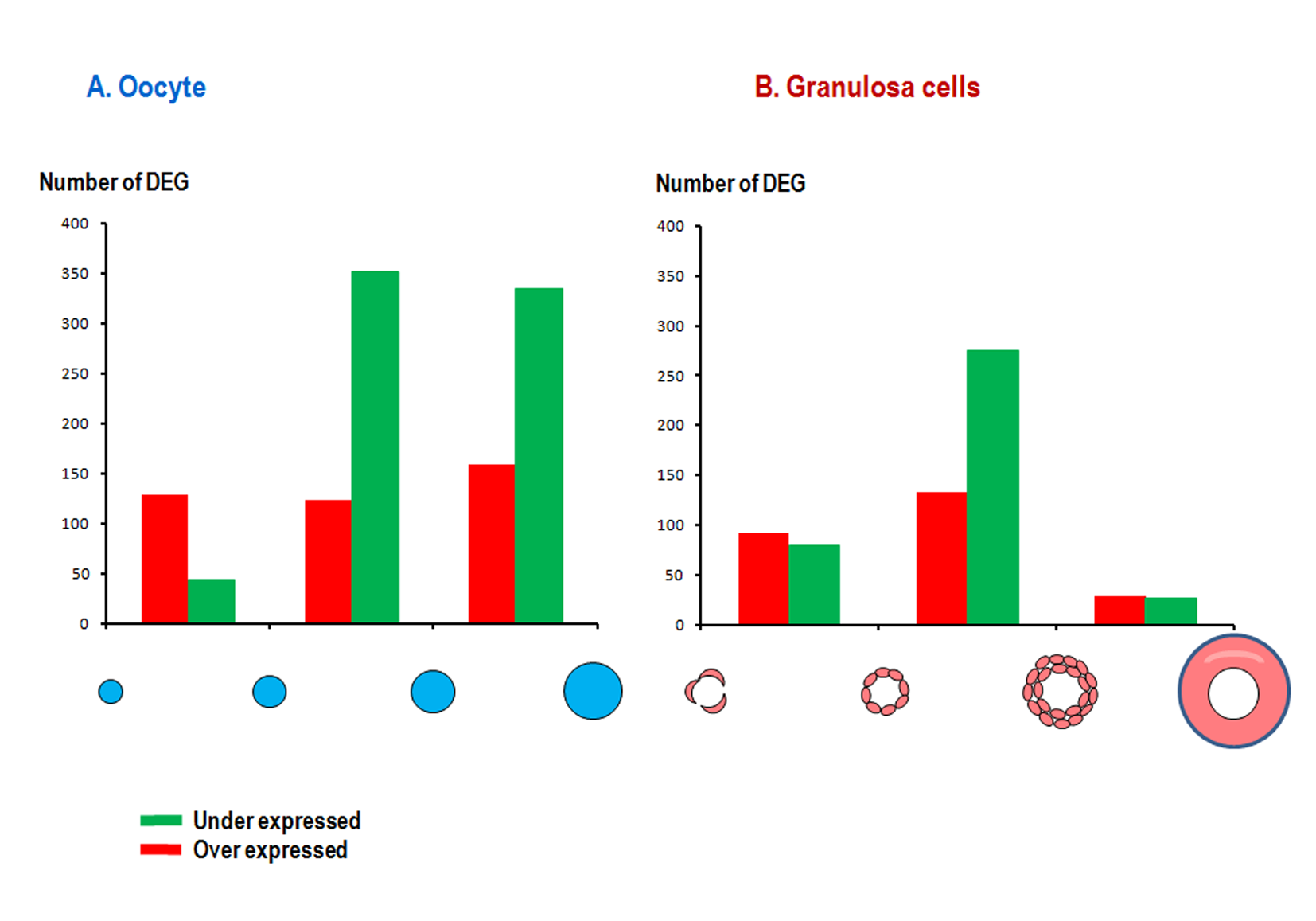

Supplement: S1 Fig — This Figure shows the number of differentially expressed genes (under- and over-expressed) in oocyte (A) and granulosa cells (B) at each follicular transition: primordial/primary, primary/secondary, secondary/small antrum. (TIF) [file pone.0141482.s001.tif]

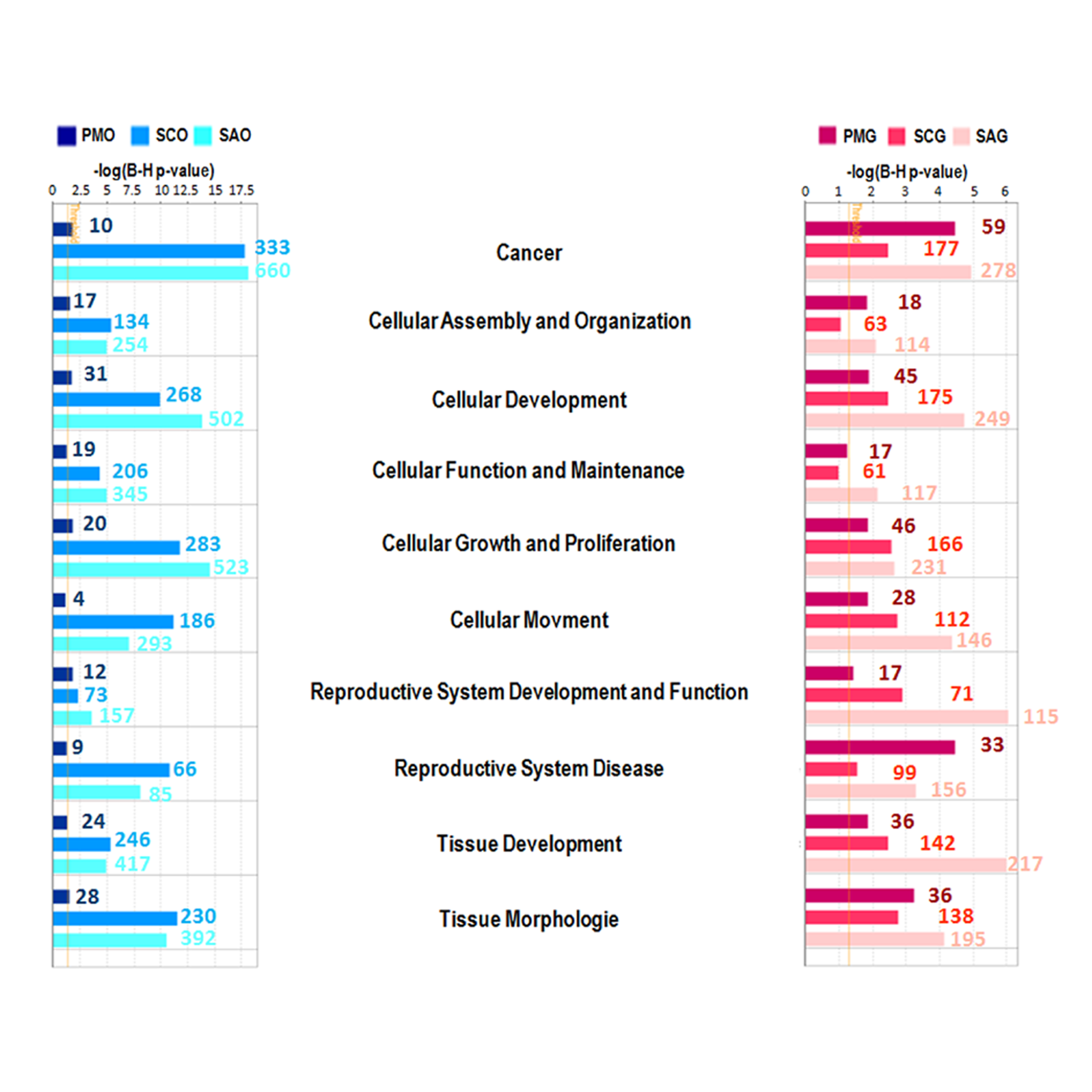

Supplement: S2 Fig — Genes differentially expressed during early development were evaluated in silico using Ingenuity Pathway Analysis (IPA) for each compartment (FDR <5%). The bar color corresponds to enriched functions at primary stage (compared to primordial stage), secondary stage (compared to primary and primordial stages), and small antrum stage (compared to secondary, primary and primordial stages). The X axis corresponds to the level of significance of the function: -log(B-H p value). Granulosa cell functions are colored in red and oocyte functions are colored in blue. Numbers correspond to the numbers of focus genes that contributed to the functions. (TIF) [file pone.0141482.s002.tif]

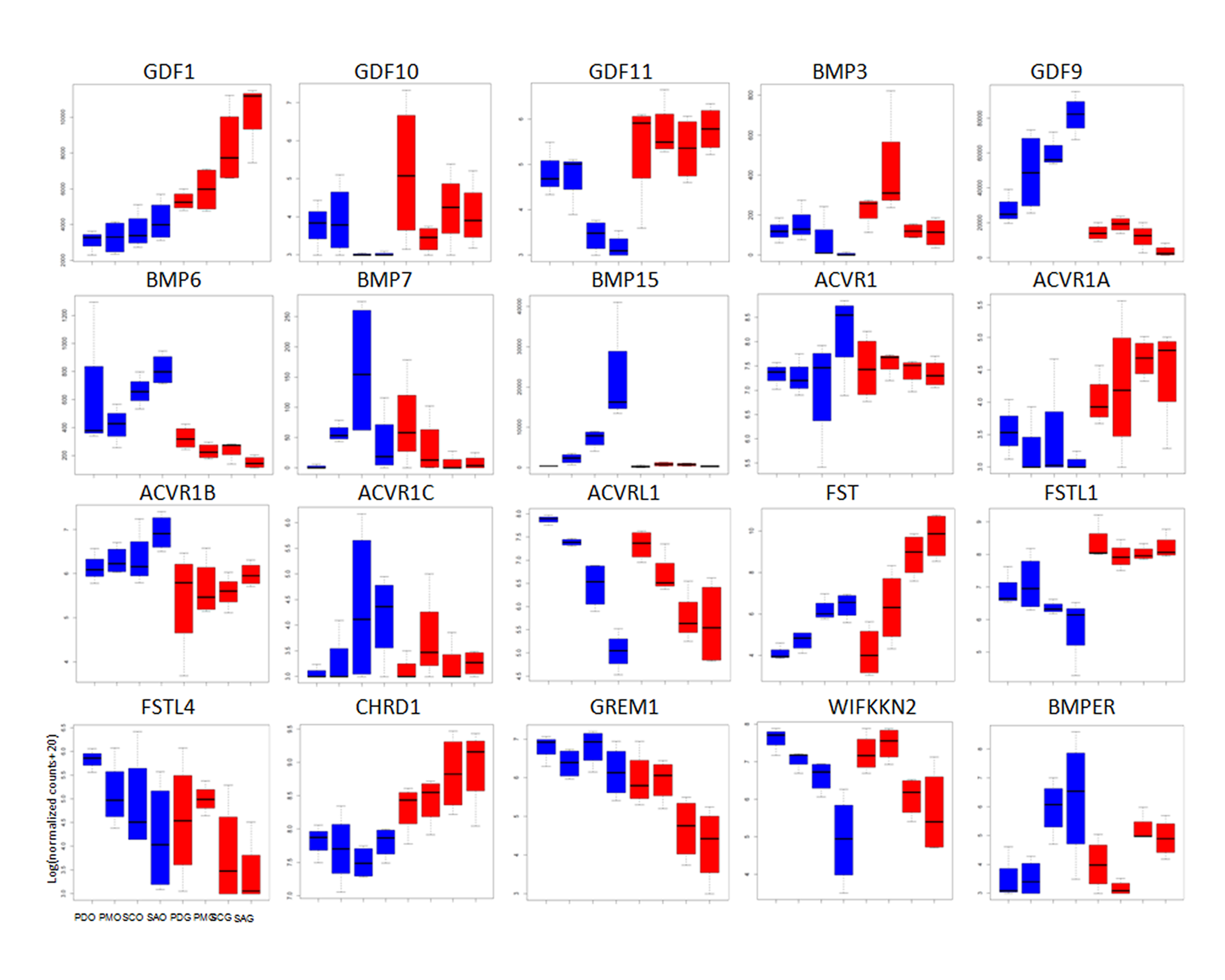

Supplement: S3 Fig — Y axis corresponds to normalized counts from RNA-seq data. GC data are colored in red and oocyte data are colored in blue. (TIF) [file pone.0141482.s003.tif]

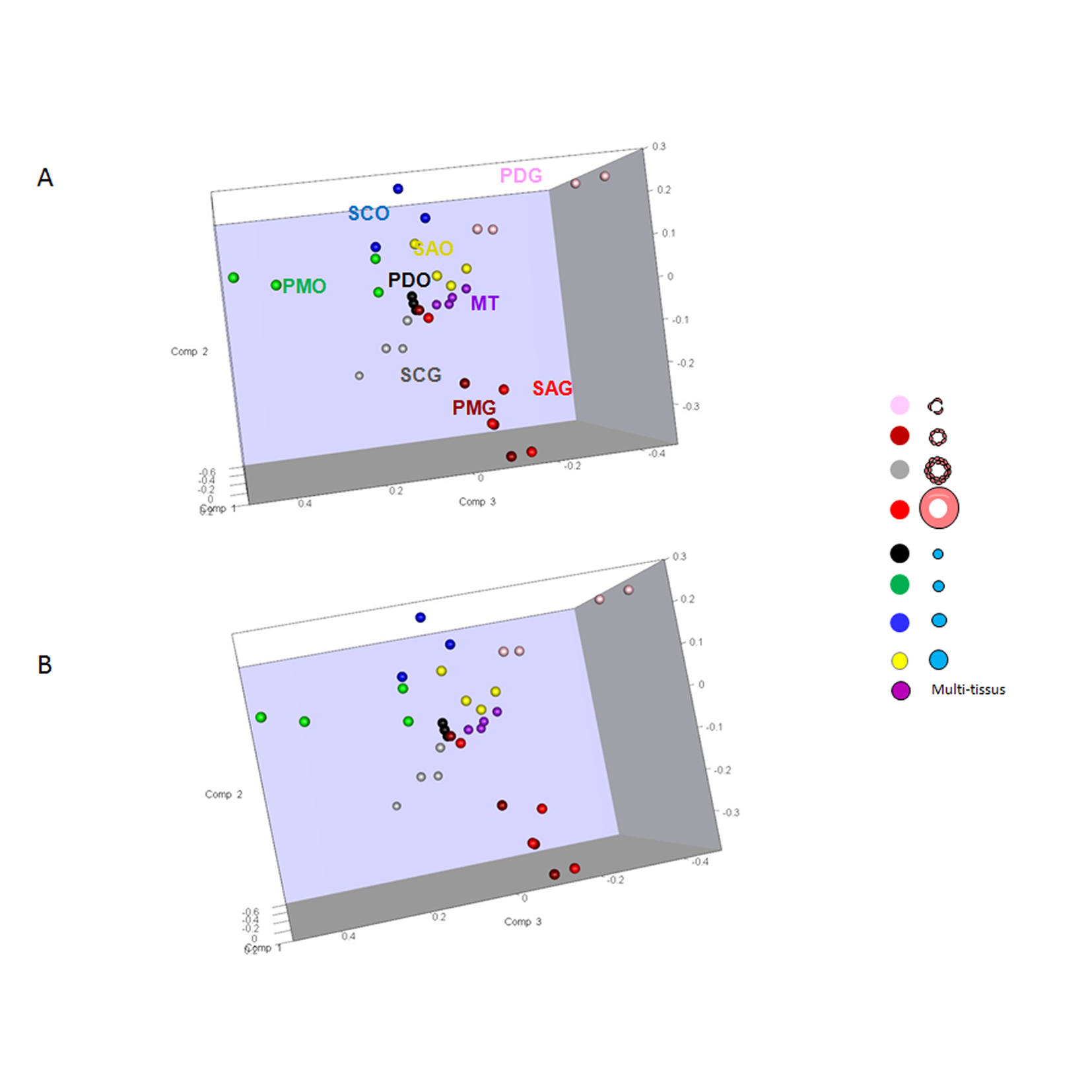

Supplement: S4 Fig — (s) PLS-DA was performed on the biomarker dataset after normalization using the DEseq R package to classify follicular stages according to gene expression. The Figure visualizes the first three components of the analysis from: (A) RNA-seq dataset, (B) qRT-PCR dataset. (TIF) [file pone.0141482.s004.tif]

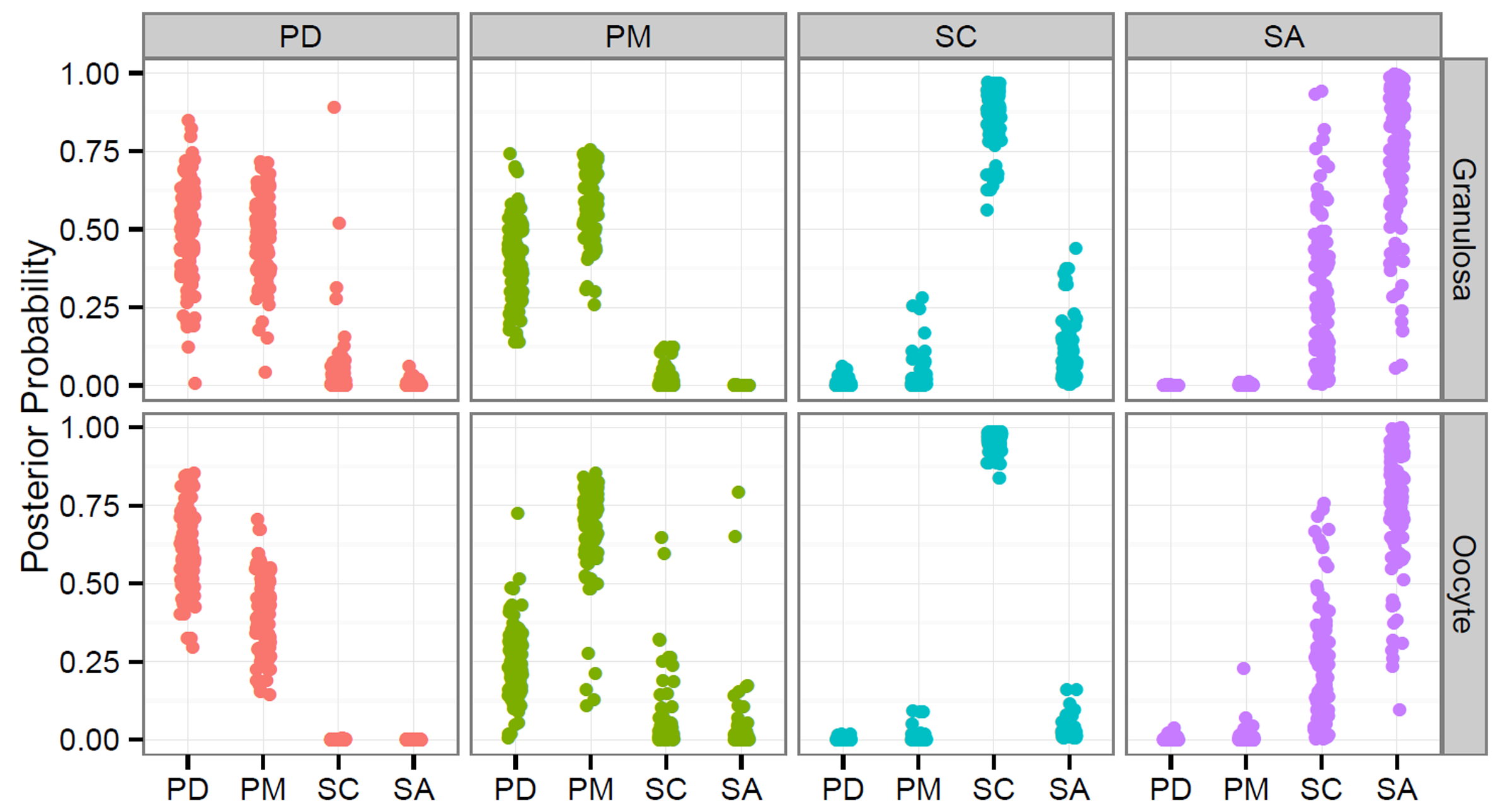

Supplement: S5 Fig — Predictive power of biomarkers using linear mixed model equations incorporating only the presence/absence of expression. The scatter-plot shows the posterior probability that an expression vector arises from each of the possible stages, when the simulated vector is made up of observations from the PD, PM, SC or SA stages (from left to right), for granulosa cells (top) and oocytes (bottom). (TIF) [file pone.0141482.s005.tif]
