# Supplementary material for: Spatio-Temporal Gene Expression Profiling during In Vivo Early Ovarian Folliculogenesis: Integrated Transcriptomic Study and Molecular Signature of Early Follicular Growth
Source: PLoS One. 2015 Nov 5;10(11):e0141482. doi: 10.1371/journal.pone.0141482 (PMC4634757; doi:10.1371/journal.pone.0141482)
Supplement: S3 Table — The expression profiles of 19 genes of interest involved in enriched canonical pathways were monitored using qRT-PCR, and statistical analysis confirmed the DE observed in the RNA-seq dataset for 14 of them. (DOCX) [file pone.0141482.s008.docx]

| **Canonical pathways** | | **Gene** | **RNAseq analysis** | | | | | | | | | | | | |  | | | **qPCR analysis** | | | | | | | | | | | | | | | | **Global effect** | | | | |
| --- | --- | --- | --- | --- | --- | --- | --- | --- | --- | --- | --- | --- | --- | --- | --- | --- | --- | --- | --- | --- | --- | --- | --- | --- | --- | --- | --- | --- | --- | --- | --- | --- | --- | --- | --- | --- | --- | --- | --- |
|  |  |  |  |  |  |  |  |  |  |  |  |  |  |  |  |  | | |  |  |  |  |  |  |  |  |  |  |  |  |  |  |  |  |  |  |  |  |  |
|  |  |  | Oocytes | | | | |  | | Granulosa cells | | | | | |  | | | Oocytes | | | | | | | |  | | | Granulosa cells | | | | | |  | | |  |
|  |  |  |  |  |  |  |  |  | |  |  |  |  |  |  |  | | |  |  |  |  |  |  |  |  |  | | |  |  |  |  |  |  |  | | |  |
|  |  |  | PM | SC | | SA | |  | | PM | | SC | | SA |  | | | PM | | | SC | | | SA | |  | | | PM | | | SC | | SA |  |  |  |  |  |
|  |  |  |  |  |  |  |  |  | |  |  |  |  |  |  | | |  |  |  |  |  |  |  |  |  | | |  |  |  |  |  |  |  |  |  |  |  |
| BMP | MAGED1 | |  | |  | | 0.26 | |  | |  |  |  | | | |  | | |  | |  |  | |  | | |  | | | |  | |  | **NS** | | |  |  |
|  | NOG | |  | | 0.002 | | 0.05 | |  | |  |  |  | | | |  | | |  | | **-** ∞ | **0.073** | |  | | |  | | | |  | |  | ****** | | |  |  |
|  | SMURF1 | |  | |  | |  | |  | |  |  | 0.05 | | | |  | | |  | |  |  | |  | | | **32.21** | | | | **0.13** | |  | ******* | | |  |  |
|  | ACVRL1 | |  | | 0.26 | | 0.05 | |  | |  |  | 0.22 | | | |  | | |  | | **0.65** |  | |  | | |  | | | |  | |  | ***** | | |  |  |
| IGF1 | IGF1 | |  | |  | | 0.11 | |  | |  |  |  | | | |  | | |  | |  |  | |  | | |  | | | |  | |  | **NS** | | |  |  |
|  | INSL3 | |  | | 17.69 | | 0.11 | |  | |  | 12.10 | 63.10 | | | |  | | |  | |  |  | |  | | |  | | | |  | | **42.25** | ***** | | |  |  |
|  | SPRY4 | |  | | 0.014 | | 579 | |  | |  |  |  | | | |  | | |  | |  |  | |  | | |  | | | |  | |  | **NS** | | |  |  |
| Gap junction | GJA1 | |  | |  | |  | |  | |  | 3.49 | 5.44 | | | |  | | |  | |  |  | |  | | |  | | | | **1.38** | | **1.50** | ***** | | |  |  |
| WNT | WIF1 | |  | |  | |  | |  | |  | 0.37 | 0.14 | | | |  | | |  | |  |  | |  | | |  | | | |  | | **0.29** | ***** | | |  |  |
|  | LRP2 | | 3.9 | | 9.11 | | 9.45 | |  | |  |  | 0.18 | | | |  | | |  | |  |  | |  | | |  | | | |  | |  | **NS** | | |  |  |
| FGF | FGF16 | |  | |  | | 4.62 | |  | | 3.03 | 0.32 | 0.14 | | | |  | | |  | |  | **4.77** | |  | | | **2.38** | | | |  | | **0.45** | ******* | | |  |  |
| PI3K | PIK3R3 | |  | |  | |  | |  | |  | 0.004 |  | | | |  | | |  | | **31.18** | **16.38** | |  | | |  | | | |  | |  | ******* | | |  |  |
|  | KITLG | |  | |  | |  | |  | |  | 3.22 | 7.09 | | | |  | | |  | |  |  | |  | | |  | | | **5.41** | | **13.32** | | ***** | |  |  |  |
|  | KIT | |  | |  | |  | |  | |  |  | 0.22 | | | |  | | |  | |  |  | |  | | |  | | | |  | |  | **NS** | | |  |  |
| RAR activation | CRABP1 | |  | | 0.11 | | 0.06 | |  | |  | 0.12 | 0.12 | | | |  | | |  | | **0.01** | **- ∞** | |  | | |  | | | |  | | **0.09** | ******* | | |  |  |
| Apoptosis | BCL2 | |  | | **- ∞** | | **+ ∞** | |  | |  |  |  | | | |  | | |  | | **0.01** | **208** | |  | | |  | | | |  | |  | ***** | | |  |  |
|  | GATA2 | |  | | 17.60 | | 78.96 | |  | |  |  |  | | | |  | | |  | |  |  | |  | | |  | | | | **- ∞** | | **+ ∞** | ***** | | |  |  |
|  | MYC | |  | |  | |  | |  | |  |  | 4.51 | | | |  | | |  | | **4.85** | **4.01** | |  | | |  | | | | **2.95** | | **3.97** | ***** | | |  |  |
|  | MOS | |  | | 3.36 | | 5.27 | |  | |  |  | 0.33 | | | |  | | |  | |  | **5.08** | |  | | |  | | | |  | | **0.23** | ******* | | |  |  |

*: p value <0.05; **: p value <0.01; ***: p value <0.005
