# Supplementary material for: Spatio-Temporal Gene Expression Profiling during In Vivo Early Ovarian Folliculogenesis: Integrated Transcriptomic Study and Molecular Signature of Early Follicular Growth
Source: PLoS One. 2015 Nov 5;10(11):e0141482. doi: 10.1371/journal.pone.0141482 (PMC4634757; doi:10.1371/journal.pone.0141482)
Supplement: S1 Text — (DOCX) [file pone.0141482.s013.docx]

### Logistic regression model for the presence/absence of expression

We first of all estimated a model based on the binary response $Y_{ijkl}$ and indicating the expression ($Y_{ijkl}=1$) or lack of expression ($Y_{ijkl}=0$), with respect to a gene $i$, developmental stage $j$, cell type $k$ and replicate $l$. This was modeled $P(Y_{ijkl}=1)$ with a hierarchial logistic regression:

$\begin{matrix} P(Y_{ijkl}=1)=\pi_{ijk} \\ \mu_{ijk}=\log(\frac{\pi_{ijk}}{1-\pi_{ijk}}) \\ \mu_{ijk}=\mu+\alpha_{j}+\beta_{k}+a_{ij}+b_{ijk} \end{matrix}$ (1)

where

$\alpha_{j}$is the stage effect on the probability of expression across all genes and cell types.

$\beta_{k}$is the cell type effect on the probability of expression across all genes and stages.

$a_{ij}$ is the random effect on the probability of expression at stage $j$ of gene $i$, with $a_{ij}:N(0,\sigma_{j}^{2})$

$b_{ijk}$is the random effect on the probability of expression at stage $j$ of cell type $k$ within gene $i$ , with $b_{ijk}:N(0,\eta_{j}^{2})$ .

The model also included correlation parameters between random effects of different stages. Model parameters were estimated by REML using the lme4 R package. All random effects were found to be highly significant ($p<10^{-7}$).

Linear regression on the quantitative level of expression

Secondly, we estimated a model on the level of expression $Z_{ijkl}$ for gene $i$, developmental stage $j$, cell type $k$ and replicate $l$. We used a fourth-root transformation of the raw expression level as response values $\mathbf{Z}$. In cases where no expression was found in any replicate, we included a single observation with null expression in the data, as the abundance of null expression was already modeled as explained above. We used a hierarchical linear regression for $E(Z_{ijkl})$ :

$\begin{matrix} E(Z_{ijk})=\mu+\alpha_{j}+\beta_{k}+a_{ij}+b_{ijk} \end{matrix}$ (2)

where:

$\alpha_{j}$is the stage effect on the level of expression across all genes and cell types.

$\beta_{k}$i is the cell type effect on the level of expression across all genes and stages.

$a_{ij}$ is the random effect on the level of expression at stage $j$ of gene $i$, with $a_{ij}:N(0,\sigma_{j}^{2})$

$b_{ijk}$ is the random effect on the level of expression at stage $j$ of cell type $k$ within gene $i$ , with $b_{ijk}:N(0,\eta_{j}^{2})$

The model also included correlation parameters between random effects of different stages. Model parameters were estimated by REML using the lme4 R package.

Equations for the prediction of developmental stage given a vector of expression data

Given the above models estimated on the RT-PCR data, it was then possible to derive equations that could predict the stage of the follicle, given the cell type and a vector of expression data on the genes. Specifically, we were dealing with a situation where we were given a vector of new observations $\tilde{\mathbf{y}}$ (binary response) and $\tilde{\mathbf{z}}$ (expression level), for a given cell type, and wanted to predict the stage reached by the corresponding follicle.

We first of all considered the new binary response data$\tilde{\mathbf{y}}$. Formally, our aim was to estimate the probability $P(X=j|\tilde{\mathbf{y}},\mathbf{y})$ that the follicle was at stage $j$ given the vector of new observation $\tilde{\mathbf{y}}$ and the original RT-PCR data $\mathbf{y}$. This probability could be written as:

where $P(X=j)$ is the *prior* probability that the follicle is in stage $j$. This typically depended on the experimental design giving rise to the new data $\tilde{\mathbf{y}}$, and at this point we placed equal prior probabilities on each possible stage. The term $P(\tilde{\mathbf{y}}|X=j,\mathbf{y})$ was the posterior predictive distribution and depended on the model parameters estimated on the initial data $\mathbf{y}$. Here we could assume that it could be factorized across genes, *i.e.* we could make the assumption that gene expressions were independent. While this assumption was probably wrong, avoiding it would require reliable quantitative knowledge on gene co-expression networks that was not available. We therefore postulated:

(3)

To compute this equation, it was necessary to be able to determine, for each gene $i$ and stage $j$, the posterior predictive distribution $P(\tilde{y}_{i}|X=j,\mathbf{y})$ that was a deterministic function of the posterior distribution of $\mu_{ijk}$. Assuming that variance components were known, *i.e.* we fixed the variance components at their estimated values, $\mu_{ijk}$ followed a gaussian distribution with mean:

and variance:

These variance terms were, for the fixed effects ($\mu$, $\alpha_{j}$, $\beta_{k}$) the variance of their estimators and for the random effects ($\tilde{a}_{ij})$ and $\tilde{b}_{ijk}$) the posterior variance of their predictor, obtained after fitting the model to the initial data $\mathbf{y}$ in R. This finally produced:

(4)

with:

(5)

These equations provided us with a first prediction of the follicular stage, after considering only the presence / absence pattern of expression in the data, *i.e.* values for the posterior probabilities $P(X=j|\tilde{\mathbf{y}},\mathbf{y})$ for each stage $j$. We then incorporated the quantitative data $\tilde{\mathbf{z}}$ as follows. It was then possible to derive a new set of prediction equations:

To compute $P(\tilde{\mathbf{z}}|X=j,\mathbf{z})$ we used the same approach as for the logistic regression above, assuming an independence between the genes and conditioning on the estimated values for variance components. For a gene $i$, we assumed that the level of expression $Z_{ijkl}$ followed a Gaussian distribution, truncated on positive real values, with mean and variance parameters calculated as above.

### Predictive ability of the models

To test the predictive ability of the models, we implemented the predictive equations using a customized program and generated new vectors for observations through re-sampling: for a given cell type and for each stage, we generated a new data vector with one observation for each gene by sampling one value taken at random from the observed data. Thus each vector was a new set of observations drawn from the original data. We performed this re-sampling method 100 times for each stage. We then applied the predictive equations to each of the 100 vectors and recorded the posterior probabilities of each stage, represented in Fig. 10 and Supplemental File 9. Supplemental File 9 shows the predictive power of the logistic regression model only (*i.e.* the above), while Fig. 10 combines the logistic regression and the linear regression model.
